# Knockdown of Inner Arm Protein IC138 in *Trypanosoma brucei* Causes Defective Motility and Flagellar Detachment

**DOI:** 10.1371/journal.pone.0139579

**Published:** 2015-11-10

**Authors:** Corinne S. Wilson, Alex J. Chang, Rebecca Greene, Sulynn Machado, Matthew W. Parsons, Taylor A. Takats, Luke J. Zambetti, Amy L. Springer

**Affiliations:** 1 Department of Biology, Siena College, Loudonville, New York, United States of America; 2 Department of Biology, Amherst College, Amherst, Massachusetts, United States of America; 3 Department of Microbiology, University of Massachusetts, Amherst, Massachusetts, United States of America; Institute of Molecular and Cell Biology, SINGAPORE

## Abstract

Motility in the protozoan parasite *Trypanosoma brucei* is conferred by a single flagellum, attached alongside the cell, which moves the cell forward using a beat that is generated from tip-to-base. We are interested in characterizing components that regulate flagellar beating, in this study we extend the characterization of TbIC138, the ortholog of a dynein intermediate chain that regulates axonemal inner arm dynein f/I1. TbIC138 was tagged *In situ*-and shown to fractionate with the inner arm components of the flagellum. RNAi knockdown of TbIC138 resulted in significantly reduced protein levels, mild growth defect and significant motility defects. These cells tended to cluster, exhibited slow and abnormal motility and some cells had partially or fully detached flagella. Slight but significant increases were observed in the incidence of mis-localized or missing kinetoplasts. To document development of the TbIC138 knockdown phenotype over time, we performed a detailed analysis of flagellar detachment and motility changes over 108 hours following induction of RNAi. Abnormal motility, such as slow twitching or irregular beating, was observed early, and became progressively more severe such that by 72 hours-post-induction, approximately 80% of the cells were immotile. Progressively more cells exhibited flagellar detachment over time, but this phenotype was not as prevalent as immotility, affecting less than 60% of the population. Detached flagella had abnormal beating, but abnormal beating was also observed in cells with no flagellar detachment, suggesting that TbIC138 has a direct, or primary, effect on the flagellar beat, whereas detachment is a secondary phenotype of TbIC138 knockdown. Our results are consistent with the role of TbIC138 as a regulator of motility, and has a phenotype amenable to more extensive structure-function analyses to further elucidate its role in the control of flagellar beat in *T*. *brucei*.

## Introduction

*Trypanosoma brucei* is a protozoan parasite found in sub Saharan Africa, where it causes sleeping sickness in humans and a related disease in other mammals, including cattle and other livestock [1]. Current drug treatments are not adequate with respect to effectiveness, safety, and expense [2,3]. There is need for better understanding of parasite biology to inform drug design, facilitate diagnosis and circumvent drug resistance [1,2,4,5]. One critical feature of *T*. *brucei* cells is motility, directed by a single flagellum that emerges from the posterior end of the cell and is attached alongside the cell body [6]. All forms of the parasite throughout its complex life cycle have motile flagella, including multiple developmental forms in both the mammalian host and the tsetse fly vector [7–9]. An unusual feature of flagellar motion of African trypanosomes is that the flagellar waveform initiates at the tip of the flagellum and propagates toward the base [10,11], this motion is thought to facilitate movement through the highly viscous extracellular environments of the mammalian host [12,13], driving the cell forwards at speeds of up to 30 μm/s [14]. Flagellar beating may not be strong enough to direct swimming against the circulatory system [14,15], but is critical for clearance of surface bound antibodies for immune evasion and may be important for parasites to efficiently negotiate complex surfaces they encounter in the blood vessels [14,16,17] and successful tissue invasion [18]. In the tsetse vector, flagellar motility is required for proper development [19].

Although the precise mechanism of motility is still being elucidated [11,13,16,20], this flagellar beat is complex and takes on a helical component as the attached flagellum rotates the cell body during swimming [11,16]. The flagellar beat can also reverse, causing tumbling and backward movement [14,21–23]. Because of these characteristics and the availability of molecular genetics and biochemical approaches, *T*. *brucei* presents an appealing model system for studying mechanisms of flagellar motility [13,24,25].

The axoneme is the skeletal structure that confers motility in eukaryotic flagella; it is highly conserved in form and protein composition [26]. The *T*. *brucei* flagellum includes an additional paracrystalline protein structure, the paraflagellar rod (PFR), that runs parallel to the axoneme along the length of the flagellum within the flagellar membrane [27] and is essential for normal flagellar motility and parasite viability in the bloodstream form [27–30]. Attachment of the flagellum to the cell body is required for proper forward motility in *T*. *brucei* [31,32]. The flagellar attachment zone (FAZ) is a specialized region of cell cortex situated along the inner leaflet of the plasma membrane with protein complexes that form interactions in the extracellular space between the plasma and flagellar membranes [33–35]. These structures are physically tethered to components of the axoneme: electron-dense filaments connect the PFR to outer arm dynein complexes on microtubule doublets 4–7 [20,33,36], and structures connecting the FAZ into the axoneme are also visible [31,37].

Aspects of axoneme structure and function have been characterized in many organisms, including the green alga *Chlamydomonas reinhardtii*, and appear to be conserved in *T*. *brucei* flagella. The structure consists of a “9+2” array: a cylindrical array of nine peripheral microtubule doublets surrounding a central pair of microtubules [38]. The microtubule doublets consist of A and B tubules, the former of which has outer arm dyneins attached along the outer rim of the axoneme, and inner arm dyneins (IAD) facing toward the inside of the axoneme [39]. The A-tubules are also connected to radial spokes that connect with the central pair complex, and nexin-dynein regulatory complexes coordinate sliding [40–42] and connect neighboring doublet pairs to one another [39,43]. Dyneins are ATP-dependent motor protein complexes that promote sliding of the A-tubule against the B-tubule of the adjacent microtubule doublet in the axoneme [44,45]. Dyneins are composed of heavy chains (HC) that contain ATPase and motor domains, as well as several intermediate chains (IC) and light chains (LC). Specific components of axonemal dynein complexes of *C*. *reinhardtii* and other organisms have been identified [26,46], and structural studies have elucidated the arrangement of dyneins along the *C*. *reinhardtii* axoneme [47–49]. The outer arm dynein complexes are arrayed 24 nm apart along the length of the A-tubule [50]. IAD complexes are more heterogeneous, repeating every 96 nm and consisting of multiple dynein species including up to seven different dynein HC [30,51]. *T*. *brucei* axonemes have similar dynein arrangements, although there are fewer individual dynein HCs in both outer and inner arm complexes [36]. The different dynein HCs in *C*. *reinhardtii* have distinct mechanical properties [26,52,53], so it is likely that they play distinct roles in coordinating proper flagellar beating [26,50,54,55]. In particular the IAD complex known as dynein f/I1, the only two-HC dynein in the inner arm, has been suggested to be essential for formation of a normal flagellar waveform by down-regulating or inhibiting microtubule sliding to modulate flagellar bending [26].

IC138, an IC of inner arm dynein f/I1, is of particular interest because it has been found to play a regulatory role in flagellar motility in *C*. *reinhardtii* [39,56–58], conveying a signal from the radial spoke/central pair pathway to dynein f/I1 [26,56,59–61]. CrIC138 assembles at the base of the two HCs and is the only phosphoprotein of dynein f/I1 [53] which, when phosphorylated, negatively regulates dynein f/I1 function [56,59,62]., Deletion of CrIC138 in results in motility defects such as reduced swimming velocities, reduced wave amplitudes and slower microtubule sliding velocities in *C*. *reinhardtii* [49,58,62,63]. Axonemes formed after CrIC138 deletion are also lacking three other proteins (IC97, LC7b and IC140) from Dynein f/I1 [49,63].

In *T*. *brucei*, orthologs of some dynein f/I1 proteins have been identified, including the two HCs, IC138 and IC140 [51]. We have previously reported that RNAi knockdown of the *T*. *brucei* ortholog, TbIC138, results in parasites with reduced motility, but no effect on overall axoneme structure [64]. In this study we extend the analysis of the motility defect. We found that TbIC138 fractionated with inner arm components of axoneme, and characterized the phenotype of the TbIC138 knockdown in terms of protein levels, cell growth, morphological changes and the timing of onset of phenotypic changes such as flagellar detachment and immotility. The motility defect was observed early after induction, and progressively became more severe. Our results show that knockdown of TbIC138 results in rapid expression of flagellar beating defects in cells with or without detached flagella, suggesting that defective flagellar beating represents a primary effect of knockdown, while detachment is a secondary phenotype.

## Materials and Methods

### Cultures and transfection

All *T*. *brucei* strains were derived from 29–13 [65], and procyclic forms were cultured using SDM-79 medium [66] with 15% heat inactivated fetal bovine serum (Invitrogen, www.lifetechnologies.com) in a 27°C CO_2_ incubator. The following antibiotics (ThermoScientific.com) were used, as appropriate: G418 15μg/mL, hygromycin 50μg/mL, phleomycin 2.5 μg/mL, puromycin (1 μg/mL), tetracycline (for dsRNA induction) 1μg/mL. Strain IC138^*RNAi*^, targeting bp 453–868 of the gene for knockdown has been described [64]. Growth was monitored by hemocytometer, viewed using a 10X objective lens in an inverted microscope; continuously growing cultures were split when they reached 6–9 x 10^6^ cells/mL to maintain log phase growth. Sedimentation analyses were performed as described [42,67].

### In situ tagging

The pMOTag system [68] was used to place a C-terminal 3xHA tag on a chromosomal copy of TbIC138 by double recombination. The 3’ end of the gene and the sequence immediately beyond the stop codon (3’UTR) were PCR-amplified using Hot Star HiFidelity Polymerase (Qiagen.com) and the following primers (IC138 3’ end: 5’ ACA CGG GCC CTG TGG TCG CCT TTC GAA CTA 3’ and 5’ ACA CGG TAC CGC CCC ACA CAT GTT TCA TGG 3’; 3’ UTR: 5’ ACA CGC GGC GCT ACC GTG CGG TAT TTA GGC 3’ and 5’ACA CTC TAG AGA TAC CCT TCC TGG TAT TGG CA 3’). For cloning, restriction sites for *Apa*I and *Kpn*1 were added to the 3’ end primer sets, and *Xba*I and *Not*I sites were added to the 3’ UTR primer sets. The respective PCR products were cloned on either side of the 3xHA tag in pMOTag2H [68]. Constructs were stably transfected into *T*. *brucei* strain 29–13 or into IC138^*RNAi*^, and clonal lines were isolated as described previously [69] to produce, respectively IC138::3xHA and IC138^*RNAi*^::3xHA.

### Quantification of knockdown

Uninduced and Tetracycline-induced IC138*i*::3x HA cultures were harvested at 48 hours- and 72 hours-post-induction (HPI), approximately 4 x 10^7^ cells were washed with cytomix [24], pelleted and resuspended in PEME buffer [70] with 1X protease inhibitor cocktail (SigmaAldrich.com), and stored in Laemmli Sample Buffer [71] for a final concentration of 2.5 x 10^5^ cells/mL. Approximately 2 x 10^6^ cell equivalents were loaded per well onto a precast Any KD acrylamide gel (Bio-Rad.com), along with markers (Precision Plus Protein Kaleidoscope, Bio-Rad). Proteins were transferred to nitrocellulose using the Trans-blot turbo transfer system (Bio-Rad), transfer was confirmed using Ponceau stain before hybridization to anti-HA- mouse monoclonal antibody, HA.11 Clone 16B12 (Covance.com) 1:1000 and anti-β-tubulin mouse monoclonal antibody, E7 (DSHB.biology.Iowa.edu) 1:5000, followed by Goat Anti-mouse horseradish peroxidase conjugate (EMDMillipore.com) 1:2500, in 1X PBS/0.05% Tween/5% nonfat dry milk. Blots were visualized using West Pico Chemiluminescent substrate (Thermo Scientific) and imaged using a ChemiDoc XRS with Quantity One software (Bio-Rad). Density of protein bands was determined using the Gel analysis/Plot Lanes tool in ImageJ (http://rsb.info.nih.gov/ij), tubulin signal in each lane was used to normalize IC138::HA from different samples. Extent of knockdown was calculated by comparing normalized IC138::HA signals from induced cultures to those of uninduced, based on three separate experiments each comprising two independent 48-hour and two independent 72-hour inductions.

### Flagellar fractionation

The two-step flagellar fractionation procedure [24,72] was used to generate samples from IC138::3xHA strain. Approximately 2 x 10^8^ cells were harvested from mid-log procyclic cultures, washed and resuspended in PEME buffer containing 1% NP-40 [1% NP-40, 100 mM piperazine-*N*,*N*′-bis(2-ethanesulfonic acid), pH 6.9, 2 mM EGTA, 0.1 mM EDTA, 1 mM MgSO_4_, 25 μg/ml aprotinin, 25 μg/ml leupeptin, 1X protease inhibitor cocktail] for 5 min at room temperature. Insoluble cytoskeletons were sedimented at 2,400 μ *g* and then washed in PMN buffer (1% NP-40, 10 mM Na_2_HPO_4_-NaH_2_PO_4_, pH 7.4, 150 mM NaCl, 1 mM MgCl_2_, 25 μg/ml aprotinin, 25 μg/ml leupeptin, 1X protease inhibitor cocktail), 0.25 mg/ml DNase I (Thermo Scientific), incubated on ice for 30 min and sedimented at 16,000 x g. Insoluble flagellar skeletons for the low salt extraction were resuspended in PMN buffer containing 500 mM NaCl (final concentration) and 0.25 mg/ml DNase I (Thermo Scientific), incubated on ice for 30 min and sedimented at 16,000 x g resulting in high salt extracted samples. All centrifugations were 10 minutes at 4°C. Six samples were collected: whole cells prior to detergent extraction (WC); insoluble cytoskeleton prior to salt extraction (Cy), intact axonemes, including outer arm components, from low salt extraction (P1), its corresponding supernatant (S1),and axonemes without outer arm components from high salt extraction (P2), and its corresponding supernatant (S2). Each sample, from two independent inductions, contained approximately 2 x 10^7^ cell equivalents and was stored in Laemmli sample buffer. Approximately 2 x 10^6^ cell equivalents were loaded per lane onto 10% TGX pre-cast SDS PAGE gel (Bio-rad.com), proteins were transferred to nitrocellulose and exposed to anti-HA and tubulin antibodies described above, or stripped with 0.1M glycine, pH2.5 and exposed to anti-trypanin mouse monoclonal antibody 37.2[42] and visualized as described above.

### Microscopy

Slides were viewed in a Nikon Eclipse Ti-S inverted microscope (NikonUSA.com), 60X objective for differential interference contrast (DIC) or epifluorescence, and a Nikon Eclipse E600 microscope, 100X objective lens for phase contrast optics. DAPI (4',6-diamidino-2-phenylindole)staining was performed as described [73], Immunofluorescence assay was described [74] for visualizing L13D6 antibody [75]. Live cells were placed in a sliding chamber prepared using tape to create a 1–2 mm space between poly-L-glutamate-treated microscope slides and coverslips [24], and observed using DIC microscopy. Flagellar lengths (signal by L13D6 antibody) and cell lengths (posterior end to flagellum tip) were measured as described [76], using ImageJ. Phase contrast images were taken using a Spot RT camera (spotimaging.com), DIC images were taken using a FLASH 2.8 CMOS camera (Hamamatsu.com). For time course analysis, at each time point approximately 100 cells were scored for degree of flagellar detachment and extent of cell motility. In cells with detached flagella, the movement of flagella themselves was scored. A scoring sheet was designed to incorporate the full spectrum of possible characteristics for each cell (S1 Table), scoring criteria and cells that could not be reliably scored are shown in S2 and S3 Tables. Statistical tests for significance were performed using MS Excel as described [77].

## Results

### Characterization of TbIC138 RNAi knockdown

Tb927.2.4060 was previously identified by Bayesian analysis [51] to be the IC138 ortholog in *T*. *brucei*. This gene encodes a predicted protein of 864 amino acids, shorter than its *C*. *reinhardtii* homolog (CrIC138) of 1057 amino acids (Genbank accession: EDP00613). Sequence analysis identified five predicted WD domains [78] in the C-terminal third of the protein, the region that has the most significant identity (37% over 353 amino acids) to CrIC138, and to the human IC138 homolog (Swiss-Prot: Q5VTH9.1) of 848 amino acids (28% identity over 683 amino acids).

**Fig 1:**
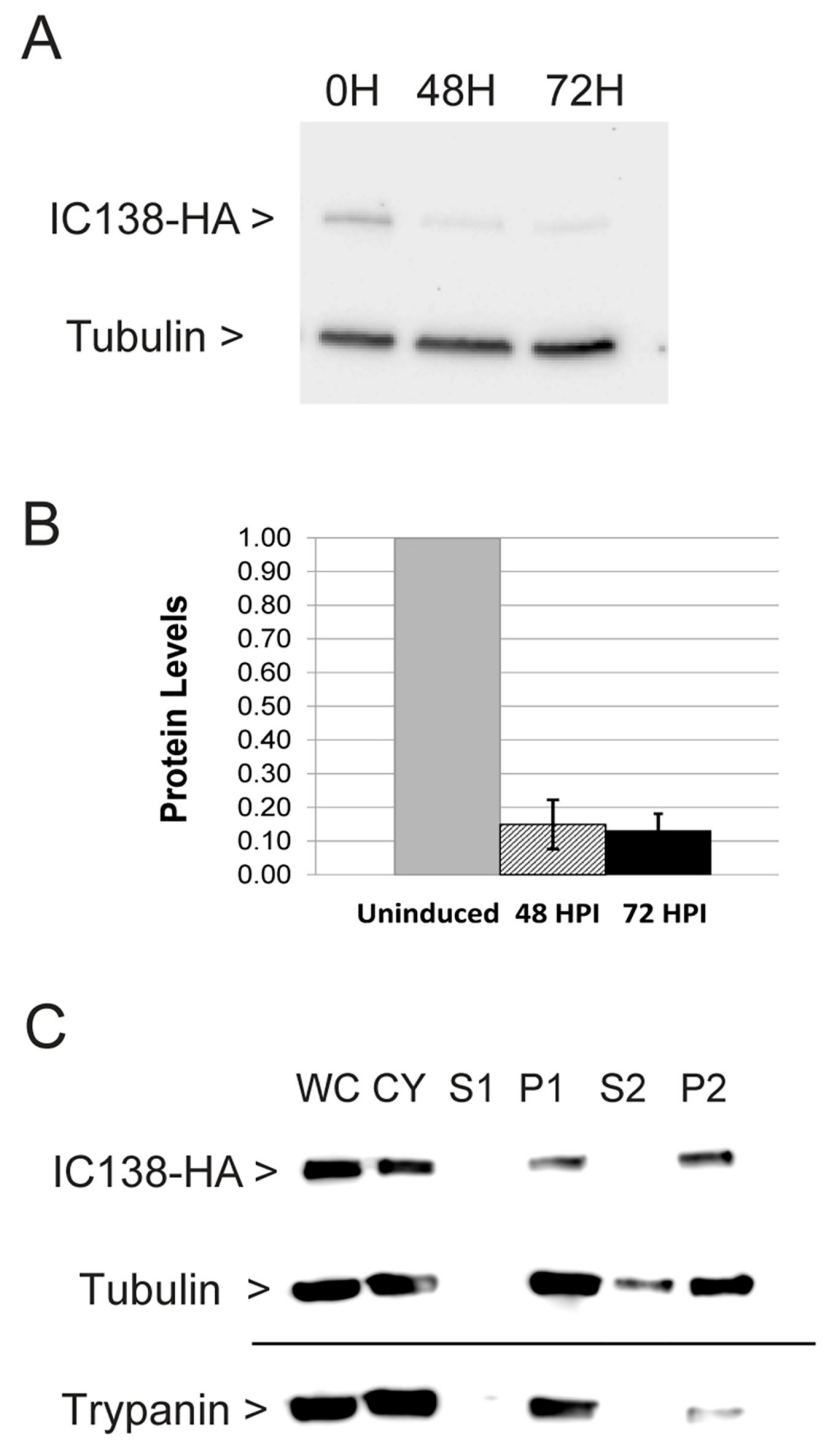
Tagged TbIC138 protein: extent of knockdown and localization. A) Western blot showing detection of IC138::3xHA (98 kD) and Tubulin (50 kD) in representative examples of IC138^*RNAi*^ cultures 0, 48 hours- and 72 hours-post-induction. B) Quantification of Western blot showing extent of knockdown of IC138 protein levels. The level of protein in uninduced cells (uninduced) was set as 1.0, and the relative expression in 48 hours- or 72 hours-post-induction cells is indicated. Data represent averages from three independent sets of uninduced and induced cultures, with standard deviation indicated by error bars. C) Western blot showing detection of IC138::3xHA, tubulin or trypanin (54 kD) in different fractions of IC138^*RNAi*^::3xHA cultures: WC = Whole cell, CY = cytoskeleton, S1,P1 = supernatant and pellet, respectively, from low salt extraction, S2, P2 = supernatant and pellet, respectively, from high salt extraction.

To study TbIC138 function, the gene was silenced using tetracycline-inducible RNAi as described previously [64]. To determine extent of knockdown of TbIC138 protein in strain IC138^*RNAi*^, we constructed an *in situ* 3x HA tag onto the C-terminal end of one chromosomal copy of the gene using the pMOtag system [68], insertion at the chromosomal copy in the IC138^*RNAI*^ strain was confirmed by PCR (not shown). Clonal IC138^*RNAi*^::3xHA was used to prepare lysates from cultures that were uninduced, or were 48 hours- or 72 hours-post-induction, respectively, using tetracycline. The resulting lysates were analyzed by western blot using anti-HA antibody to detect tagged TbIC138, and anti-tubulin antibody for normalization (Fig 1A). The predicted size of IC138::3xHA is 98 kD, and a band of this size was readily detectable.

Quantification of band intensities revealed IC138::3xHA protein levels were reduced to 15% of uninduced levels at 48 hours-post-induction, or 13% of uninduced at 72 hours-post-induction (Fig 1B). This suggests that the knockdown of TbIC138 protein is substantial.

### Localization

IC138::3xHA can also be used to confirm that this protein is actually localized in the IAD fraction in *T*. *brucei*. Clonal lines of IC138::3xHA were used to prepare lysates from various steps in flagellar preparation. The resulting fractions were analyzed by western blot using anti-HA antibody to detect tagged protein of the appropriate size in various fractions (Fig 1C). A band of the correct size was readily detectable in low- and high- salt insoluble flagellar fractions (P1, P2), indicating that TbIC138 is not extracted with high salt, as expected of an inner arm protein. Antibodies against two other proteins with known localization were used as controls [79], both exhibited fractionation patterns as expected: β-tubulin is a cytoskeletal protein and was found in cytoskeletal and insoluble flagellar fractions as well as in the S2 fraction [80]. Trypanin localizes at the base of the inner arm and was found in low- and high-salt insoluble flagellar fractions but not in supernatant fractions [81]. Control samples prepared from the untransfected host strain 29–13 yielded no anti-HA signal in any fractions (S5 Fig).

### Growth and sedimentation

**Fig 2:**
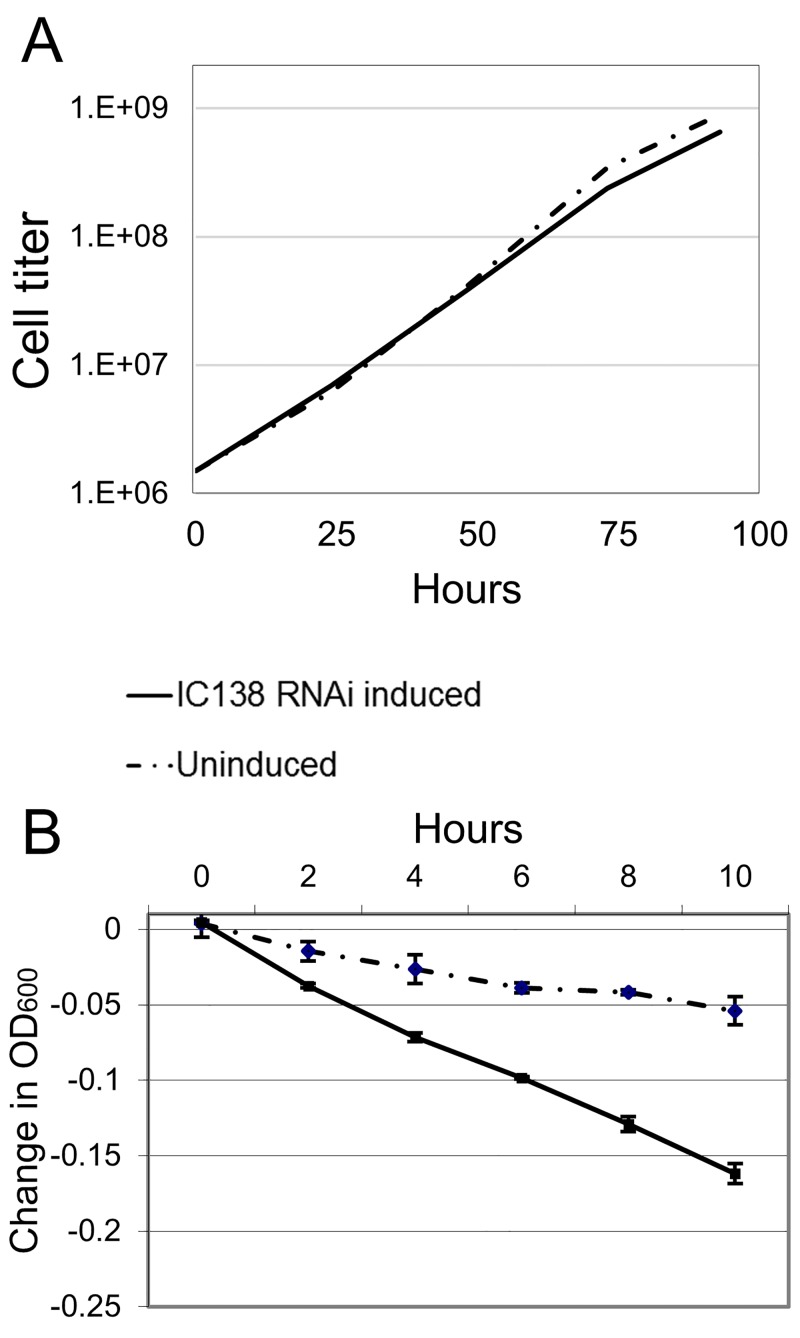
Growth and sedimentation analyses of TbIC138 knockdown. A) A representative cumulative growth curve of IC138^*RNAi*^ uninduced (dashed line) or tetracycline-induced (solid line). Cell titer = cells/mL. B) Sedimentation assay showing the motility of IC138^*RNAi*^ cells in uninduced (dashed line) or induced (solid line) by monitoring absorbance at 600nm for a 10-hour period starting at 48 hours-post-induction. Error bars indicate standard deviation between replicate vials.

RNAi knockdown of TbIC138 resulted in a slight reduction in growth rate (Fig 2A) observable within 48 hours-post-induction. Individual cells became noticeably less motile and clusters of three or more cells were visible. Cell growth did not appear to stop even after inductions of as many as 8 days (S1 Fig), but as cell clusters became larger and more frequent, it is possible that clumped cells could cause an underestimate of cell titers at later times post-induction. Mild agitation of cultures for the duration of the growth curve analysis did not seem to affect the growth rate difference (S1 Fig) and did not eliminate clustering behavior. Sedimentation analysis of IC138^*RNAi*^ showed that induced cells settled out of solution more rapidly than uninduced cells, representing defective cell motility (Fig 2B).

### Effect on cell cycle

**Fig 3:**
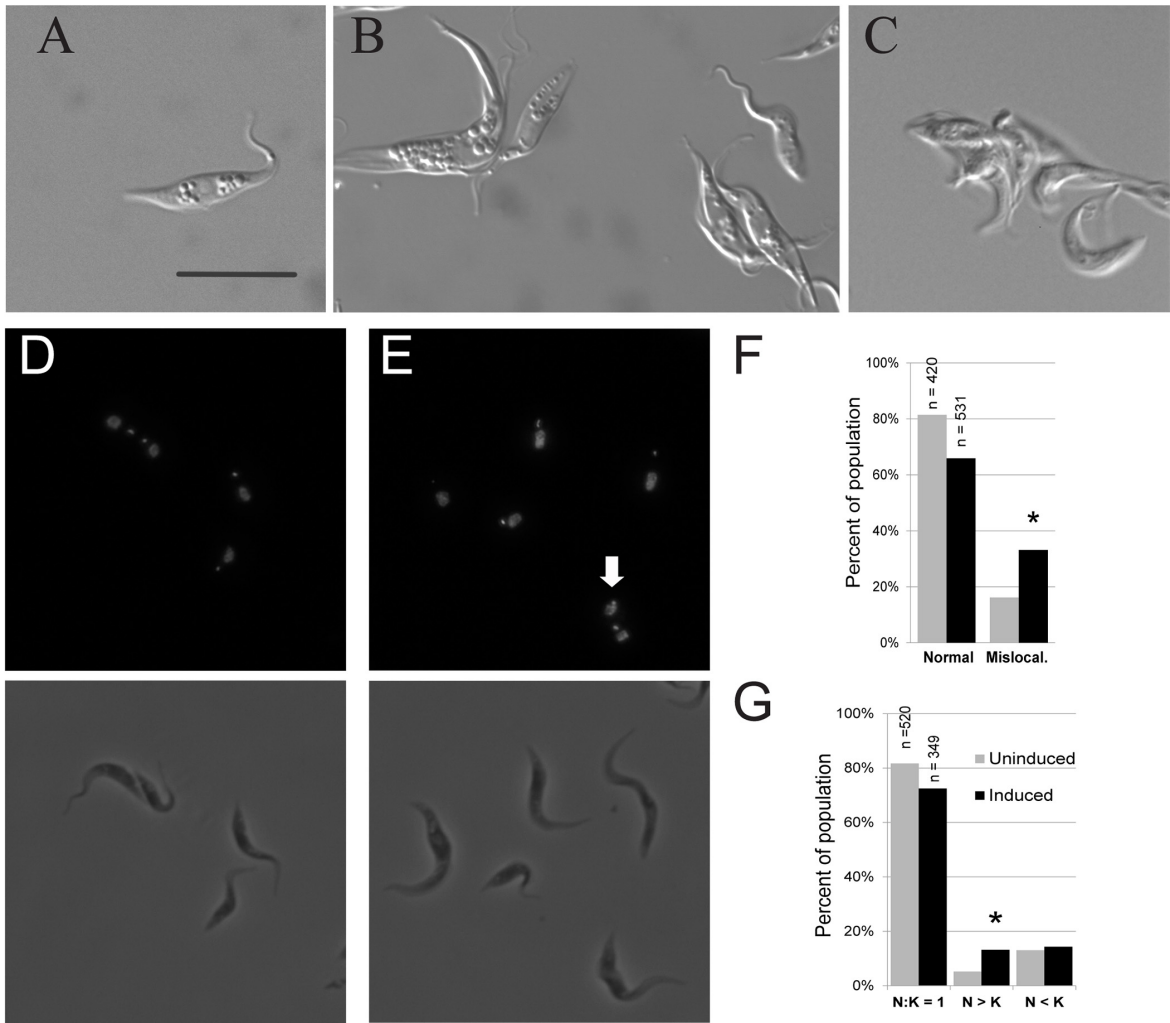
Cell cycle defects in TbIC138 knockdown. DIC, fluorescent and phase contrast images of IC138^*RNAi*^ cells: A) Individual live cell from uninduced culture. B) Individual live cell and clusters of live cells in 48 hours-post-induction cultures. C) Live cell cluster from 72 hours-post-induction culture. Paired DAPI-stained and phase contrast images of D) uninduced IC138^*RNAi*^ cells, and E) IC138^*RNAi*^ cells 48 hours-post-induction. White arrowhead indicates mis-localized kinetoplast. F) Graph showing percentages of normally localized (gray) and mis-localized (black) kinetoplasts in uninduced and 48 hours-post-induction cultures of IC138^*RNAi*^. Asterisks indicate significance, number of samples (n) is indicated above bar. Scale bars represent 10 μm. G) Graph showing percentages of occurrence of different N:K ratios in uninduced and 48 hours-post-induction cultures of IC138^*RNAi*^.

Microscopy was used to observe abnormalities associated with defects in cell growth and cell cycle progression in induced IC138^*RNAi*^ cells compared to uninduced cultures (Fig 3A). By 48 hours-post-induction, several clusters of multiple cells were visible (Fig 3B) and these increased in frequency and number of cells through the course of induction, often the number of cells was difficult to classify (Fig 3C). In some cases flagella in these clusters appeared to be entangled, and some clusters seemed to have more one flagellum per cell body. Small clusters were occasionally seen in uninduced IC138^*RNAi*^ cultures, but much less frequently. The cell cycle in *T*. *brucei* includes replication of nuclear and kinetoplast DNA, and defects affecting cell cycle progression can result in abnormal nucleus:kinetoplast (N:K) ratios or kinetoplast localization. We used DAPI to stain nuclei and kinetoplasts in both uninduced and 48 hours-post-induction IC138^*RNAi*^ cultures. At 48 hours-post-induction, the mean number of nuclei per cell in IC138^*RNAi*^ was 1.20 and the mean for uninduced cultures was 1.13 (S4 Table), a difference that was not significant by single factor analysis of variance (P < 0.001). In IC138^*RNAi*^ cultures 48 hours post-induction kinetoplasts were more likely to be mis-localized (Fig 3D and 3E), generally by being located closer to the nucleus than expected (roughly one third or less of the average distance) or not discernable. Approximately 33% of the induced IC138^*RNAi*^ cells had mis-localized kinetoplasts at 48 hours-post-induction, as compared to 16% of uninduced (Fig 3F), this difference was shown to be significant by both Chi Squared and Fisher’s exact tests for independence (P< 0.001). Occasionally, kinetoplasts were found anterior to the nucleus (S2 Fig), this was observed less often in the induced IC138^*RNAi*^ cultures but the difference was not significant. 48 hours-post-induction cultures also showed a 13% incidence of cells with N:K ratios greater than 1, compared to 5% in uninduced cultures (Fig 3G and S2 Fig), this difference was significant by both Chi Squared and Fisher’s exact tests for independence (P< 0.001). The frequency of cells with N:K ratios less than 1 was 14% for induced cultures and 13% for uninduced cultures, a difference that was not significant.

### Morphological features

**Fig 4:**
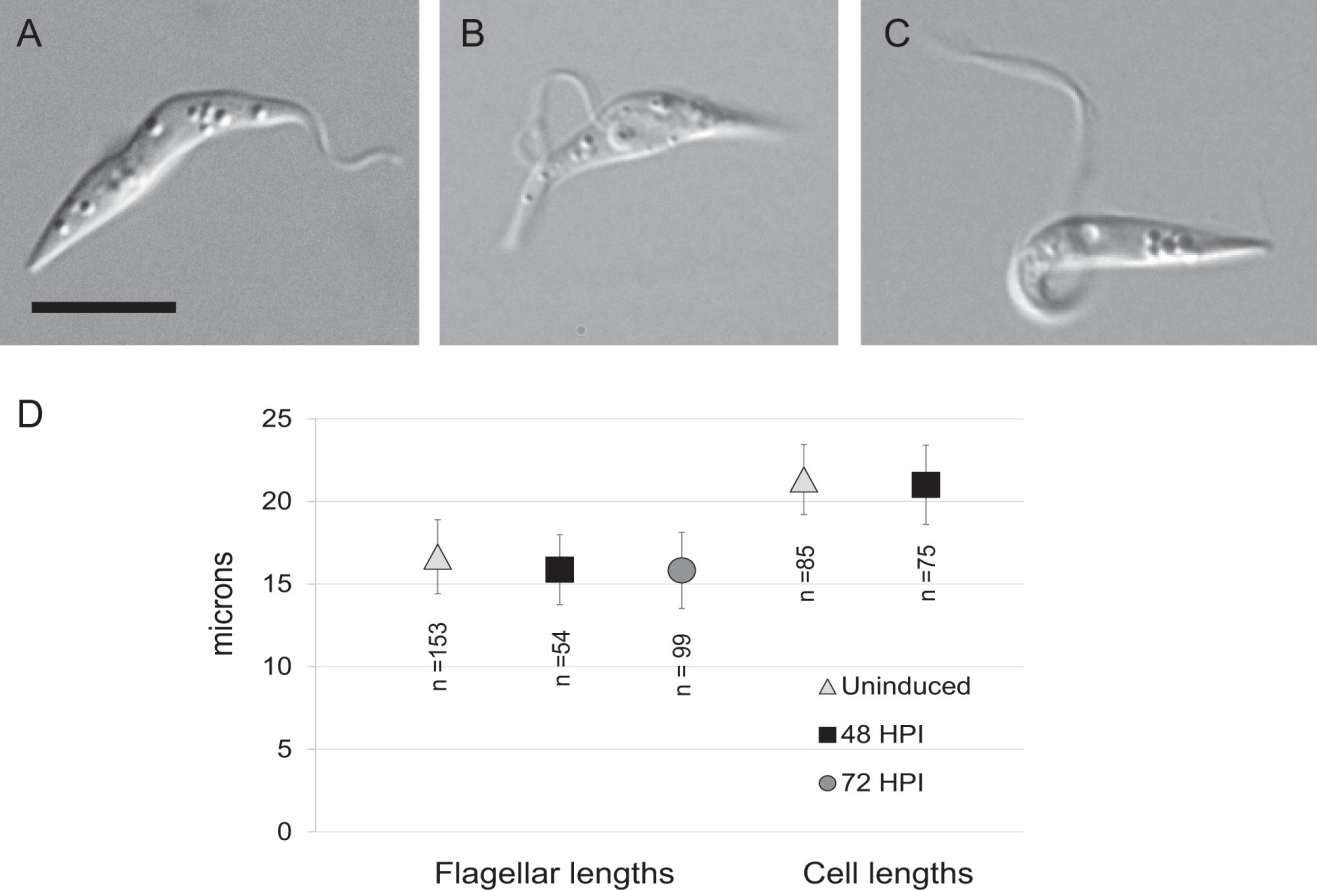
Morphological features of TbIC138 knockdown. DIC images showing examples of attached (A), partially detached (B) and fully detached (C) flagella. Scale bar represents 5 μm. D) Graph showing flagellar lengths and total cell lengths of uninduced, 48 hours- and 72 hours-post-induction IC138^*RNAi*^ cells. Error bars show standard deviation, number of samples (n) is indicated below each point.

We used microscopy to examine individual IC138^*RNAi*^ induced cells for changes in flagellar structure and cell size. Depletion of flagellar proteins has been shown to affect flagellar morphology [6], including the attachment to the cell body. Partially detached flagella were defined as those in which the flagellum was attached to the cell body at the flagellar pocket and at the anterior end, but had a portion in between that is disconnected, forming a loop. Fully detached flagella were attached only at the flagellar pocket and were not in contact with any other part of the cell body. Cells with either partially or fully detached flagella were observed in IC138^*RNAi*^ induced cultures. Fig 4 shows examples of a cell with completely attached flagellum (Fig 4A), a partially detached flagellum (Fig 4B) and with fully detached flagellum (Fig 4C). We also measured lengths of flagella, as well as total cell length in uninduced and induced IC138^*RNAi*^ cultures. Flagellar length was measured using anti-PFR antibody L13D6, and cell length was measured from phase contrast images (Fig 4D). While the mean flagellar length in 48 hours-post-induction and (15.87 μm) 72 hours-post-induction (15.83 μm) cultures was shorter than in uninduced (16.66 μm) IC138^*RNAi*^ cultures, the variance in length was relatively high and the difference in mean lengths was not significant using 2-tailed unpaired t tests (P>0.001). Cell length was measured from microscopic images of cells containing one nucleus/one kinetoplast. No significant difference was observed between mean cell lengths from IC138^*RNAi*^ uninduced cultures (21.33 μm) and those from 48 hours-post-induction cultures (21.01 μm).

### Development of phenotype over time

To observe the time course of the knockdown phenotype, cells were scored for 108 hours at 12 hour time points following induction of RNAi. One of the aspects of this phenotype is that over time more and more cells were found in clusters of three or more cells, and by later time points some of these clusters contained upwards of ten cells. These clusters could be broken up to some extent by mechanical agitation (i.e shaking flasks by hand rapidly for 2–3 seconds), but then new clusters formed within 24 hours. This clustering caused some technical difficulties in obtaining reliable observations of characteristics of induced IC138^*RNAi*^ cells. Care was taken to vigorously agitate cultures before sampling to separate cells, and pipetting was used to further enrich for separation, although occasionally clusters were observed on microscope slides (S3 Table). Some cells that appeared to be stuck to the surface of the slide were eliminated from scoring; if too many cells appeared to be stuck a new slide was prepared. For consistency, only individual cells for which motility and flagellar attachment could be definitively scored were counted.

**Fig 5:**
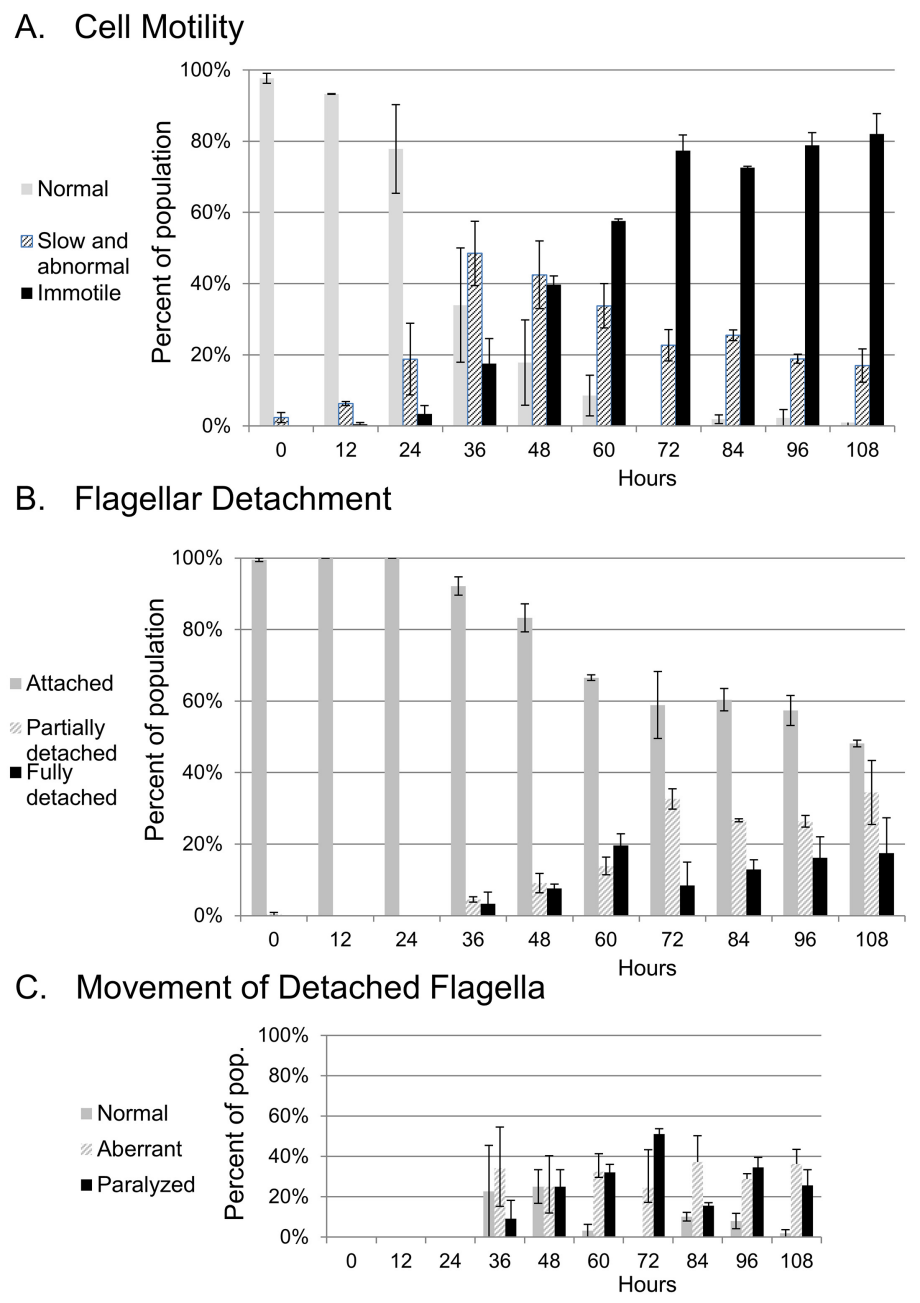
Occurrence of phenotypes over time following knockdown of TbIC138. A) Frequency of cells with normal, slow and abnormal motility or immotile. See S2 Table for motility classification. B) Frequency of cells with attached, partially detached or fully detached flagella. C) Frequency of detached flagella showing normal movement, aberrant movement or paralysis. Error bars represent standard deviation between two independent experiments, keys are at left of each graph.

The results presenting development of abnormal motility and flagellar detachment are shown in Fig 5. By 24 hours-post-induction, some abnormal motility was observed (Fig 5A). Cells scored as slow or abnormal included those with slowed rotation or pulsating body movements, as well as cells that twitched irregularly (these behaviors were combined into one category: “slow and abnormal motility”). Abnormal motility was generally initiated tip-to-base, even in cells that were twitching or very slow. However, we did not specifically score the direction of beat in each cell in this study, so we cannot rule out the occasional occurrence of a reverse beat within this phenotype. Cells scored as immotile had no body movement but usually still had some flagellar beating, either at the flagellar tip or along detached portions of the flagellum. Cells that showed no movement at all of any part of the body or flagellum were very rare, and could not be distinguished from non-living cells. Motility became steadily more defective over the time course of the study. Cells exhibiting slow and abnormal movement increased as a proportion of the population for 36 hours-post-induction, then decreased and the percentage of immotile cells increased. By 108 hours-post-induction more than 80% of cells were immotile. Abnormal movement was rarely observed in uninduced cultures (S3 Fig).

Flagellar detachment was first observed somewhat later than abnormal motility, by the 36 hours-post-induction time point, and partial detachment was generally more prevalent than complete detachment. The percentage of affected cells increased over time and by 108 hours-post-induction, cells with partially or fully detached flagella comprised nearly 60% of the population (Fig 5B). Flagella that were detached, either partially or fully, usually showed defective beating (Fig 5C and S4 Fig) although generally less than 50% of detached flagella showed complete paralysis (no detectable movement). Detached flagella were very rare in the uninduced cultures (S3 Fig), but were occasionally observed. Since we made a point of using uninduced IC138^*RNAi*^ as our reference control instead of a wild type strain, our controls may show a low frequency of phenotypes associated with flagellar defects if repression of dsRNA transcription was leaky in the absence of tetracycline.

## Discussion

In this study we have examined TbIC138, ortholog of an IAD intermediate chain protein believed to be a regulator of flagellar motility [49,56,58]. We confirmed that RNAi knockdown of TbIC138 results in substantially reduced protein levels using an *in situ* 3xHA tag at the 3’ end of a chromosomal copy of the gene. Western analyses confirmed that IC138::3xHA is found in flagellar fractions (Fig 1C) and in particular, in the insoluble fraction following high salt extraction (P2). In this fraction the outer arm dyneins are missing and central pair proteins are significantly reduced, but IADs largely remain [24]. The localization of TbIC138 is consistent with that of IAD structures [81], as would be expected of an IC138 ortholog.

Knockdown of TbIC138 results in defective motility [64]; in this study we have extended the characterization of the motility defect using sedimentation and microscopic analyses to show that IC138^*RNAi*^ induced cells sedimented at a faster rate than uninduced cells (Fig 2B) and that cell movement was progressively more impaired over time following induction, such that nearly 80% of cells were immotile by 72 hours-post-induction (Fig 5). Cell growth showed only a slight reduction in rate upon induction of RNAi (Fig 2A), and cells did not stop growing even after 8 days (S1 Fig). TbIC138 knockdown resulted in formation of clusters of cells, noticeable within 48 hours-post-induction (Fig 3B and 3C); mechanical agitation could be used to separate cells but the clustering behavior continued upon subsequent culturing. Clustering is a feature that has been observed following RNAi against other flagellar proteins [21,42,74], and may indicate that the final stages of cytokinesis do not function properly in these strains.

To assess whether the knockdown affected other aspects of cell cycle function, nuclear and kinetoplast characteristics were measured, such as multinucleate cells, high N:K ratios and missing or mis-localized kinetoplasts. All of these parameters increase in incidence upon knockdown of another component of dynein f/I1, the alpha HC TbDNAH10 [74]. In IC138^RNAi^ 48 hours-post-induction, the number of multinucleate cells was not significantly increased relative to uninduced cultures (S2 Table), but there were slight yet significant increases in cells with N:K > 1 and in cells with mis-localized kinetoplasts (Fig 3F and 3G). The increase in N:K ratio can result from kinetoplasts that overlapped the nucleus in the image and so were blocked from view, which may be a result of mis-localization. Disruption of cell cycle events can lead to changes in cell and flagellar length [31,82–85], but we found no differences in these lengths following TbIC138 knockdown. Taken together, these results may suggest a mild effect on timing of kinetoplast division and/or kinetoplast segregation. Previous reviews have discussed the occurrence of cell cycle defects associated with depletion of flagellar proteins in *T*. *brucei* [6,86], and suggest motility may play a role in the completion of cell division. The phenotypes we report here, with respect to growth rate and N:K ratio were milder defects than those seen after knockdown of TbDNAH10, even though extent of knockdown was similar [74]. It is perhaps not surprising that depletion of this protein, thought to modulate dynein function, does not have as drastic an impact on cell growth and movement as depletion of a dynein HC [58,74].

To document the time course of development of the IC138^*RNAi*^ phenotype, we monitored cells at 12 hour intervals following induction of RNAi. We focused on features of flagellar detachment and motility that could be easily scored in live cells. While some features were limited to only a small number of cells, the study does reveal some consistent trends: discernable changes in cell motion were recorded as early as 24 hours-post-induction (Fig 5A), and motility became more defective over time until nearly all cells were immotile. It remains to be determined if the increase in immotility over time was related to the dynamics of assembly and recycling of axonemal complexes following knockdown, or resulted from loss of proper coordination between axonemal dyneins.

Incidence of flagellar detachment was first observed a little bit later (36 hours-post-induction), partially detached flagella were more common than completely detached flagella, but both populations increased in frequency over time (Fig 5B). By 108 hours nearly 60% of cells showed some form of flagellar detachment. Detachment of flagella from along the cell is not unique to the TbIC138 knockdown, it has been reported in studies of other flagellar and cell cycle factors, [21,86–89], and there may be many ways to compromise attachment.

Attachment of the flagellum to the cell body occurs at the FAZ, a structure demarcated by an electron-dense filament that includes multi-domain protein assemblies [6,35,36,90], including FLAM3 that attaches into the axoneme itself [31]. Proper positioning of a new flagellum is important for the progression of cell division, including kinetoplast localization [82,91–94]. Failure to properly attach results in shorter cells and significant morphological changes [31,83,85,90], unlike the phenotype seen upon knockdown of TbIC138. Depletion of TbIC138 was previously shown to not affect axoneme structure by transmission electron microscopic analysis [64] that shows the major IAD structures are still intact despite the detachment phenotype.

The mechanism by which TbIC138 depletion leads to flagellar detachment is unknown, but it is possible that irregular beating of flagella pulls apart the attachment anchoring complexes, resulting in progressively more flagellar detachment over time, but we were not able to observe individual cells long enough to determine this. Alternatively, there may be components that interact with anchoring complexes whose assembly into the flagellum is compromised after TbIC138 knockdown [63,95], resulting in weaker flagellum-cell body attachment.

Flagella with partial or complete detachment nearly always had abnormal beating (Fig 5C), that appeared as a slow and often irregular beat, or as complete paralysis. Because the numbers of cells with detached flagella were low, it was not possible to determine by the end of this study if all detached flagella would eventually become paralyzed, although it seems likely since immotility increases in the population overall. By characterizing cells with respect to flagellar attachment and separately to body movement, we are able to make distinctions between behavior of cells with attached and detached flagella. Clearly, depletion of TbIC138 can affect flagellar beating in the absence of flagellar detachment, indeed when development of abnormal motility over time is considered in only those cells with completely attached flagella, the pattern is essentially the same (S4 Fig) as for the total cell population (Fig 5A). Since we are interested in regulation of flagellar beating in *T*. *brucei*, monitoring the course of phenotype development allowed us to determine time points that might be useful for analyses of flagellar beating specifically. Since the earliest patterns of abnormal motility were seen by 24 hours-post-induction, detailed aspects of flagellar beating patterns can be analyzed shortly after induction without concern that secondary phenotypes associated with cell cycle timing will interfere with the analysis. Alternatively, later time points in which individual cells with fully detached flagella can be observed could be studied to observe variations in beating patterns of free flagella, comparing patterns between time points, or relative to a strain such as the FLAM3 knockdown strain that has detached flagella with normal motility [31].

In genome-wide studies, depletion of any of several IAD proteins are reported to result in loss of fitness in bloodstream form parasites [30,96], suggesting that components of IAD play important roles in parasite survival in mammalian hosts. Among IAD proteins, IC138 is an important regulator [49,56], recent studies have suggested that IC138 functions to position the dynein f/I1 complex properly in *C*. *reinhardtii* [26,97]. The phenotype observed for knockdown of TbIC138 represents a stronger motility defect than seen in *C*. *reinhardtii*, in which loss of CrIC38 does not cause immotility [26], but a less severe motility defect than that of knockdown of a dynein f/I1 HC, consistent with its role as a regulator, as stated above. Differences in flagellar motility between the two organisms may result in slightly different roles for dynein f/I1 and thus regulators like IC138. The TbIC138 knockdown resulted in a distinct defect in flagellar beating, suggesting this strain is amenable for more extensive structure -function analyses of the role of this important protein in *T*. *brucei*. Flagellar motility is important not only in protozoan pathogens [12], but in many eukaryotic organisms, where it plays a role in cell movement but also in the sensory and transduction functions of this organelle [25].

## Supporting Information

S1 Fig Additional Growth curves. Following RNAi induction for 8 days (A), and comparing Agitation Vs. Stationary incubation (B). For agitation, flasks were incubated on an orbital shaker at 80 rpm for the duration of the induction.
(PDF)

S2 Fig Complete breakdown of Nucleus: Kinetoplast ratios and kinetoplast localization. N:K classes (A) and location of kinetoplasts, including frequency of anterior localization (B).
(PDF)

S3 Fig Phenotype scoring for uninduced IC138^*RNAi*^ cultures. Scoring for cell motility phenotypes (A) and for flagellar detachment phenotypes (B).
(PDF)

S4 Fig Additional phenotypic comparisons for induced IC138^*RNAi*^. Motility in IC138^*RNAi*^ induced cells that have partially or fully detached flagella (A) and cell body movement in IC138^*RNAi*^ induced cells, only those with completely attached flagella (B).
(PDF)

S5 Fig Images of complete Western Blots used for Fig 1. A. Paired images of blot used for knockdown quantification (Fig 1A). At left, a photograph of Ponceau-stained blot with pre-stained molecular weight markers indicated and at right, chemiluminescent signal from digital imager. Lanes are as follows: 1: Molecular weight marker, 2: Uninduced, 3: 48 H (replicate 1), 4: 48 H (repl. 2), 5: 72 H (repl. 1), 6: 72 H (repl. 2), 7: Uninduced, 8: 48 H (repl. 1), 9: 72 H (repl. 1). B. Matched images of blot used for detection of IC138::3xHA in fractions (Fig 1C), a photograph of Ponceau-stained blot with pre-stained molecular weight markers visible (left), chemiluminescent signal from immunoblot using anti-HA and anti-tubulin (center) and with anti-trypanin (right) from digital imager. Lanes are as follows: 1: Positive control for IC138::3xHA signal, 2: Molecular weight marker, 3: WC, 4: CY, 5: S1, 6: P1, 7: S2, 8: P2. C. Scanned image of film used for detection of IC138::3xHA in both IC138::3xHA strain and 29–13 negative control strain. Location of markers indicated after alignment of blot and film. Lanes are as follows: 1: Molecular weight marker, 2–5: IC138::3xHA in HS, LS, CY and WC fractions, respectively. 6–9: Wild type strain 29–13 in HS, LS, CY, and WC, respectively. 10: Positive control for HA epitope.
(PDF)

S1 Table Scoring sheet for flagellar detachment and cell motility phenotypes. (PDF)

S2 Table Definitions of phenotypic characteristics used in scoring sheet. (DOCX)

S3 Table Frequencies of cells that couldn’t be scored or of large clusters. (XLSX)

S4 Table Nucleus: Cell ratios for IC138^*RNAi*^ induced and uninduced cultures. (PDF)

## References

[1] Cnops J, Magez S, DE Trez C. Escape mechanisms of African trypanosomes: why trypanosomosis is keeping us awake. Parasitology. 2014; 1–11. 10.1017/S0031182014001838 25479093

[2] Baker N, de Koning HP, Mäser P, Horn D. Drug resistance in African trypanosomiasis: the melarsoprol and pentamidine story. Trends Parasitol. 2013;29: 110–118. 10.1016/j.pt.2012.12.005 23375541 PMC3831158

[3] Franco J, Simarro P, Diarra, Ruiz Postigo, Jannin. Diversity of human African trypanosomiasis epidemiological settings requires fine-tuning control strategies to facilitate disease elimination. Res Rep Trop Med. 2013; 1 10.2147/RRTM.S40157 PMC6067614 30100778

[4] Brun R, Blum J. Human African trypanosomiasis. Infect Dis Clin North Am. 2012;26: 261–273. 10.1016/j.idc.2012.03.003 22632638

[5] Pays E, Vanhollebeke B, Uzureau P, Lecordier L, Pérez-Morga D. The molecular arms race between African trypanosomes and humans. Nat Rev Microbiol. 2014;12: 575–584. 10.1038/nrmicro3298 24975321

[6] Ralston KS, Kabututu ZP, Melehani JH, Oberholzer M, Hill KL. The Trypanosoma brucei flagellum: moving parasites in new directions. Annu Rev Microbiol. 2009;63: 335–362. 10.1146/annurev.micro.091208.073353 19575562 PMC3821760

[7] Vickerman K. Developmental cycles and biology of pathogenic trypanosomes. Br Med Bull. 1985;41: 105–114. 3928017 10.1093/oxfordjournals.bmb.a072036

[8] Matthews KR. The developmental cell biology of Trypanosoma brucei. J Cell Sci. 2005;118: 283–290. 10.1242/jcs.01649 15654017 PMC2686837

[9] Van Den Abbeele J, Claes Y, van Bockstaele D, Le Ray D, Coosemans M. Trypanosoma brucei spp. development in the tsetse fly: characterization of the post-mesocyclic stages in the foregut and proboscis. Parasitology. 1999;118 (Pt 5): 469–478. 10363280 10.1017/s0031182099004217

[10] Gadelha C, Wickstead B, Gull K. Flagellar and ciliary beating in trypanosome motility. Cell Motil Cytoskeleton. 2007;64: 629–643. 10.1002/cm.20210 17549738

[11] Rodríguez JA, Lopez MA, Thayer MC, Zhao Y, Oberholzer M, Chang DD, et al Propulsion of African trypanosomes is driven by bihelical waves with alternating chirality separated by kinks. Proc Natl Acad Sci U S A. 2009;106: 19322–19327. 10.1073/pnas.0907001106 19880745 PMC2780783

[12] Langousis G, Hill KL. Motility and more: the flagellum of Trypanosoma brucei. Nat Rev Microbiol. 2014;12: 505–518. 10.1038/nrmicro3274 24931043 PMC4278896

[13] Stellamanns E, Uppaluri S, Hochstetter A, Heddergott N, Engstler M, Pfohl T. Optical trapping reveals propulsion forces, power generation and motility efficiency of the unicellular parasites Trypanosoma brucei brucei. Sci Rep. 2014;4: 6515 10.1038/srep06515 25269514 PMC4180810

[14] Heddergott N, Krüger T, Babu SB, Wei A, Stellamanns E, Uppaluri S, et al Trypanosome motion represents an adaptation to the crowded environment of the vertebrate bloodstream. PLoS Pathog. 2012;8: e1003023 10.1371/journal.ppat.1003023 23166495 PMC3499580

[15] Wheeler RJ, Gluenz E, Gull K. The limits on trypanosomatid morphological diversity. PloS One. 2013;8: e79581 10.1371/journal.pone.0079581 24260255 PMC3834336

[16] Weiße S, Heddergott N, Heydt M, Pflästerer D, Maier T, Haraszti T, et al A quantitative 3D motility analysis of Trypanosoma brucei by use of digital in-line holographic microscopy. PloS One. 2012;7: e37296 10.1371/journal.pone.0037296 22629379 PMC3358310

[17] Engstler M, Pfohl T, Herminghaus S, Boshart M, Wiegertjes G, Heddergott N, et al Hydrodynamic flow-mediated protein sorting on the cell surface of trypanosomes. Cell. 2007;131: 505–515. 10.1016/j.cell.2007.08.046 17981118

[18] Vickerman K, Tetley L, Hendry KA, Turner CM. Biology of African trypanosomes in the tsetse fly. Biol Cell Auspices Eur Cell Biol Organ. 1988;64: 109–119. 10.1016/0248-4900(88)90070-6 3067793

[19] Rotureau B, Ooi C-P, Huet D, Perrot S, Bastin P. Forward motility is essential for trypanosome infection in the tsetse fly. Cell Microbiol. 2014;16: 425–433. 10.1111/cmi.12230 24134537

[20] Koyfman AY, Schmid MF, Gheiratmand L, Fu CJ, Khant HA, Huang D, et al Structure of Trypanosoma brucei flagellum accounts for its bihelical motion. Proc Natl Acad Sci U S A. 2011;108: 11105–11108. 10.1073/pnas.1103634108 21690369 PMC3131312

[21] Branche C, Kohl L, Toutirais G, Buisson J, Cosson J, Bastin P. Conserved and specific functions of axoneme components in trypanosome motility. J Cell Sci. 2006;119: 3443–3455. 10.1242/jcs.03078 16882690

[22] Baron DM, Ralston KS, Kabututu ZP, Hill KL. Functional genomics in Trypanosoma brucei identifies evolutionarily conserved components of motile flagella. J Cell Sci. 2007;120: 478–491. 10.1242/jcs.03352 17227795

[23] Uppaluri S, Nagler J, Stellamanns E, Heddergott N, Herminghaus S, Engstler M, et al Impact of microscopic motility on the swimming behavior of parasites: straighter trypanosomes are more directional. PLoS Comput Biol. 2011;7: e1002058 10.1371/journal.pcbi.1002058 21698122 PMC3116898

[24] Oberholzer M, Lopez MA, Ralston KS, Hill KL. Approaches for functional analysis of flagellar proteins in African trypanosomes. Methods Cell Biol. 2009;93: 21–57. 10.1016/S0091-679X(08)93002-8 20409810 PMC3821762

[25] Vincensini L, Blisnick T, Bastin P. 1001 model organisms to study cilia and flagella. Biol Cell Auspices Eur Cell Biol Organ. 2011;103: 109–130. 10.1042/BC20100104 21275904

[26] Kamiya R, Yagi T. Functional diversity of axonemal dyneins as assessed by in vitro and in vivo motility assays of chlamydomonas mutants. Zoolog Sci. 2014;31: 633–644. 10.2108/zs140066 25284382

[27] Bastin P, Sherwin T, Gull K. Paraflagellar rod is vital for trypanosome motility. Nature. 1998;391: 548 10.1038/35300 9468133

[28] Bastin P, MacRae TH, Francis SB, Matthews KR, Gull K. Flagellar morphogenesis: protein targeting and assembly in the paraflagellar rod of trypanosomes. Mol Cell Biol. 1999;19: 8191–8200. 10567544 10.1128/mcb.19.12.8191 PMC84903

[29] Santrich C, Moore L, Sherwin T, Bastin P, Brokaw C, Gull K, et al A motility function for the paraflagellar rod of Leishmania parasites revealed by PFR-2 gene knockouts. Mol Biochem Parasitol. 1997;90: 95–109. 9497035 10.1016/s0166-6851(97)00149-7

[30] Broadhead R, Dawe HR, Farr H, Griffiths S, Hart SR, Portman N, et al Flagellar motility is required for the viability of the bloodstream trypanosome. Nature. 2006;440: 224–227. 10.1038/nature04541 16525475

[31] Rotureau B, Blisnick T, Subota I, Julkowska D, Cayet N, Perrot S, et al Flagellar adhesion in Trypanosoma brucei relies on interactions between different skeletal structures in the flagellum and cell body. J Cell Sci. 2014;127: 204–215. 10.1242/jcs.136424 24163437

[32] LaCount DJ, Bruse S, Hill KL, Donelson JE. Double-stranded RNA interference in Trypanosoma brucei using head-to-head promoters. Mol Biochem Parasitol. 2000;111: 67–76. 11087917 10.1016/s0166-6851(00)00300-5

[33] Sherwin T, Gull K. The cell division cycle of Trypanosoma brucei brucei: timing of event markers and cytoskeletal modulations. Philos Trans R Soc Lond B Biol Sci. 1989;323: 573–588. 2568647 10.1098/rstb.1989.0037

[34] Rocha GM, Brandão BA, Mortara RA, Attias M, de Souza W, Carvalho TMU. The flagellar attachment zone of Trypanosoma cruzi epimastigote forms. J Struct Biol. 2006;154: 89–99. 10.1016/j.jsb.2005.11.008 16414276

[35] Höög JL, Bouchet-Marquis C, McIntosh JR, Hoenger A, Gull K. Cryo-electron tomography and 3-D analysis of the intact flagellum in Trypanosoma brucei. J Struct Biol. 2012;178: 189–198. 10.1016/j.jsb.2012.01.009 22285651 PMC3355306

[36] Hughes LC, Ralston KS, Hill KL, Zhou ZH. Three-Dimensional Structure of the Trypanosome Flagellum Suggests that the Paraflagellar Rod Functions as a Biomechanical Spring. PLoS ONE. 2012;7 10.1371/journal.pone.0025700 22235240 PMC3250385

[37] Sun SY, Wang C, Yuan YA, He CY. An intracellular membrane junction consisting of flagellum adhesion glycoproteins links flagellum biogenesis to cell morphogenesis in Trypanosoma brucei. J Cell Sci. 2013;126: 520–531. 10.1242/jcs.113621 23178943

[38] Summers KE, Gibbons IR. Adenosine triphosphate-induced sliding of tubules in trypsin-treated flagella of sea-urchin sperm. Proc Natl Acad Sci U S A. 1971;68: 3092–3096. 5289252 10.1073/pnas.68.12.3092 PMC389597

[39] Porter ME, Sale WS. The 9 + 2 Axoneme Anchors Multiple Inner Arm Dyneins and a Network of Kinases and Phosphatases That Control Motility. J Cell Biol. 2000;151: 37–42. 10.1083/jcb.151.5.f37 PMC2174360 11086017

[40] Hutchings NR, Donelson JE, Hill KL. Trypanin is a cytoskeletal linker protein and is required for cell motility in African trypanosomes. J Cell Biol. 2002;156: 867–877. 10.1083/jcb.200201036 11864997 PMC2173309

[41] Piperno G, Mead K, LeDizet M, Moscatelli A. Mutations in the “dynein regulatory complex” alter the ATP-insensitive binding sites for inner arm dyneins in Chlamydomonas axonemes. J Cell Biol. 1994;125: 1109–1117. 8195292 10.1083/jcb.125.5.1109 PMC2120054

[42] Ralston KS, Lerner AG, Diener DR, Hill KL. Flagellar Motility Contributes to Cytokinesis in Trypanosoma brucei and Is Modulated by an Evolutionarily Conserved Dynein Regulatory System. Eukaryot Cell. 2006;5: 696–711. 10.1128/EC.5.4.696-711.2006 16607017 PMC1459671

[43] Heuser T, Raytchev M, Krell J, Porter ME, Nicastro D. The dynein regulatory complex is the nexin link and a major regulatory node in cilia and flagella. J Cell Biol. 2009;187: 921–933. 10.1083/jcb.200908067 20008568 PMC2806320

[44] King SM. Integrated control of axonemal dynein AAA(+) motors. J Struct Biol. 2012;179: 222–228. 10.1016/j.jsb.2012.02.013 22406539 PMC3378790

[45] Ueno H, Bui KH, Ishikawa T, Imai Y, Yamaguchi T, Ishikawa T. Structure of dimeric axonemal dynein in cilia suggests an alternative mechanism of force generation. Cytoskelet Hoboken NJ. 2014;71: 412–422. 10.1002/cm.21180 24953776

[46] Inaba K. Molecular basis of sperm flagellar axonemes: structural and evolutionary aspects. Ann N Y Acad Sci. 2007;1101: 506–526. 10.1196/annals.1389.017 17363437

[47] Bui KH, Sakakibara H, Movassagh T, Oiwa K, Ishikawa T. Molecular architecture of inner dynein arms in situ in Chlamydomonas reinhardtii flagella. J Cell Biol. 2008;183: 923–932. 10.1083/jcb.200808050 19029338 PMC2592835

[48] Nicastro D, Schwartz C, Pierson J, Gaudette R, Porter ME, McIntosh JR. The molecular architecture of axonemes revealed by cryoelectron tomography. Science. 2006;313: 944–948. 10.1126/science.1128618 16917055

[49] Heuser T, Barber CF, Lin J, Krell J, Rebesco M, Porter ME, et al Cryoelectron tomography reveals doublet-specific structures and unique interactions in the I1 dynein. Proc Natl Acad Sci U S A. 2012;109: E2067–2076. 10.1073/pnas.1120690109 22733763 PMC3409752

[50] Ishikawa T, Sakakibara H, Oiwa K. The architecture of outer dynein arms in situ. J Mol Biol. 2007;368: 1249–1258. 10.1016/j.jmb.2007.02.072 17391698

[51] Wickstead B, Gull K. Dyneins across eukaryotes: a comparative genomic analysis. Traffic Cph Den. 2007;8: 1708–1721. 10.1111/j.1600-0854.2007.00646.x PMC2239267 17897317

[52] Bayly PV, Lewis BL, Kemp PS, Pless RB, Dutcher SK. Efficient spatiotemporal analysis of the flagellar waveform of Chlamydomonas reinhardtii. Cytoskelet Hoboken NJ. 2010;67: 56–69. 10.1002/cm.20424 PMC4109274 20169530

[53] Kotani N, Sakakibara H, Burgess SA, Kojima H, Oiwa K. Mechanical properties of inner-arm dynein-f (dynein I1) studied with in vitro motility assays. Biophys J. 2007;93: 886–894. 10.1529/biophysj.106.101964 17496036 PMC1913158

[54] Brokaw CJ, Kamiya R. Bending patterns of Chlamydomonas flagella: IV. Mutants with defects in inner and outer dynein arms indicate differences in dynein arm function. Cell Motil Cytoskeleton. 1987;8: 68–75. 10.1002/cm.970080110 2958145

[55] Shimizu Y, Sakakibara H, Kojima H, Oiwa K. Slow Axonemal Dynein e Facilitates the Motility of Faster Dynein c. Biophys J. 2014;106: 2157–2165. 10.1016/j.bpj.2014.04.009 24853744 PMC4052281

[56] Wirschell M, Hendrickson T, Sale WS. Keeping an eye on I1: I1 dynein as a model for flagellar dynein assembly and regulation. Cell Motil Cytoskeleton. 2007;64: 569–579. 10.1002/cm.20211 17549744

[57] King SJ, Dutcher SK. Phosphoregulation of an inner dynein arm complex in Chlamydomonas reinhardtii is altered in phototactic mutant strains. J Cell Biol. 1997;136: 177–191. 9008712 10.1083/jcb.136.1.177 PMC2132467

[58] VanderWaal KE, Yamamoto R, Wakabayashi K, Fox L, Kamiya R, Dutcher SK, et al bop5 Mutations reveal new roles for the IC138 phosphoprotein in the regulation of flagellar motility and asymmetric waveforms. Mol Biol Cell. 2011;22: 2862–2874. 10.1091/mbc.E11-03-0270 21697502 PMC3154882

[59] Habermacher G, Sale WS. Regulation of flagellar dynein by phosphorylation of a 138-kD inner arm dynein intermediate chain. J Cell Biol. 1997;136: 167–176. 9008711 10.1083/jcb.136.1.167 PMC2132463

[60] Oda T, Yanagisawa H, Yagi T, Kikkawa M. Mechanosignaling between central apparatus and radial spokes controls axonemal dynein activity. J Cell Biol. 2014;204: 807–819. 10.1083/jcb.201312014 24590175 PMC3941055

[61] Hendrickson TW, Goss JL, Seaton CA, Rohrs HW. The IC138 and IC140 intermediate chains of the I1 axonemal dynein complex bind directly to tubulin. Biochim Biophys Acta. 2013;1833: 3265–3271. 10.1016/j.bbamcr.2013.09.011 24080090

[62] Hendrickson TW, Perrone CA, Griffin P, Wuichet K, Mueller J, Yang P, et al IC138 is a WD-repeat dynein intermediate chain required for light chain assembly and regulation of flagellar bending. Mol Biol Cell. 2004;15: 5431–5442. 10.1091/mbc.E04-08-0694 15469982 PMC532023

[63] Bower R, VanderWaal K, O’Toole E, Fox L, Perrone C, Mueller J, et al IC138 Defines a Subdomain at the Base of the I1 Dynein That Regulates Microtubule Sliding and Flagellar Motility. Mol Biol Cell. 2009;20: 3055–3063. 10.1091/mbc.E09-04-0277 19420135 PMC2704157

[64] Zukas R, Chang AJ, Rice M, Springer AL. Structural analysis of flagellar axonemes from inner arm dynein knockdown strains of Trypanosoma brucei. Biocell Off J Soc Latinoam Microsc Electron Al. 2012;36: 133–141. 23682429

[65] Wirtz E, Leal S, Ochatt C, Cross GA. A tightly regulated inducible expression system for conditional gene knock-outs and dominant-negative genetics in Trypanosoma brucei. Mol Biochem Parasitol. 1999;99: 89–101. 10215027 10.1016/s0166-6851(99)00002-x

[66] Brun R, Schönenberger. Cultivation and in vitro cloning or procyclic culture forms of Trypanosoma brucei in a semi-defined medium. Short communication. Acta Trop. 1979;36: 289–292. 43092

[67] Bastin P, Pullen TJ, Sherwin T, Gull K. Protein transport and flagellum assembly dynamics revealed by analysis of the paralysed trypanosome mutant snl-1. J Cell Sci. 1999;112 (Pt 21): 3769–3777. 10523512 10.1242/jcs.112.21.3769

[68] Oberholzer M, Morand S, Kunz S, Seebeck T. A vector series for rapid PCR-mediated C-terminal in situ tagging of Trypanosoma brucei genes. Mol Biochem Parasitol. 2006;145: 117–120. 10.1016/j.molbiopara.2005.09.002 16269191

[69] Chandler J, Vandoros AV, Mozeleski B, Klingbeil MM. Stem-loop silencing reveals that a third mitochondrial DNA polymerase, POLID, is required for kinetoplast DNA replication in trypanosomes. Eukaryot Cell. 2008;7: 2141–2146. 10.1128/EC.00199-08 18849470 PMC2593185

[70] Oberholzer M, Lopez MA, Ralston KS, Hill KL. Approaches for functional analysis of flagellar proteins in African trypanosomes. Methods Cell Biol. 2009;93: 21–57. 10.1016/S0091-679X(08)93002-8 20409810 PMC3821762

[71] Laemmli UK. Cleavage of structural proteins during the assembly of the head of bacteriophage T4. Nature. 1970;227: 680–685. 5432063 10.1038/227680a0

[72] Robinson D, Beattie P, Sherwin T, Gull K. Microtubules, tubulin, and microtubule-associated proteins of trypanosomes. Methods Enzymol. 1991;196: 285–299. 2034124 10.1016/0076-6879(91)96027-o

[73] Wheeler RJ, Gull K, Gluenz E. Detailed interrogation of trypanosome cell biology via differential organelle staining and automated image analysis. BMC Biol. 2012;10: 1 10.1186/1741-7007-10-1 22214525 PMC3398262

[74] Springer AL, Bruhn DF, Kinzel KW, Rosenthal NF, Zukas R, Klingbeil MM. Silencing of a putative inner arm dynein heavy chain results in flagellar immotility in Trypanosoma brucei. Mol Biochem Parasitol. 2011;175: 68–75. 10.1016/j.molbiopara.2010.09.005 20888370 PMC2974060

[75] Kohl L, Sherwin T, Gull K. Assembly of the paraflagellar rod and the flagellum attachment zone complex during the Trypanosoma brucei cell cycle. J Eukaryot Microbiol. 1999;46: 105–109. 10361731 10.1111/j.1550-7408.1999.tb04592.x

[76] Rotureau B, Subota I, Bastin P. Molecular bases of cytoskeleton plasticity during the Trypanosoma brucei parasite cycle. Cell Microbiol. 2011;13: 705–716. 10.1111/j.1462-5822.2010.01566.x 21159115

[77] McDonald JH. Handbook of Biological Statistics Third Sparky House Publishing, Baltimore MD; 2014.

[78] Neer EJ, Schmidt CJ, Nambudripad R, Smith TF. The ancient regulatory-protein family of WD-repeat proteins. Nature. 1994;371: 297–300. 10.1038/371297a0 8090199

[79] Ralston KS, Kisalu NK, Hill KL. Structure-Function Analysis of Dynein Light Chain 1 Identifies Viable Motility Mutants in Bloodstream-Form Trypanosoma brucei. Eukaryot Cell. 2011;10: 884–894. 10.1128/EC.00298-10 21378260 PMC3147417

[80] Hill KL, Hutchings NR, Grandgenett PM, Donelson JE. T lymphocyte-triggering factor of african trypanosomes is associated with the flagellar fraction of the cytoskeleton and represents a new family of proteins that are present in several divergent eukaryotes. J Biol Chem. 2000;275: 39369–39378. 10.1074/jbc.M006907200 10969087

[81] Ralston KS, Hill KL. Trypanin, a Component of the Flagellar Dynein Regulatory Complex, Is Essential in Bloodstream Form African Trypanosomes. PLoS Pathog. 2006;2. 10.1371/journal.ppat.0020101 PMC1579245 17009870

[82] Kohl L, Robinson D, Bastin P. Novel roles for the flagellum in cell morphogenesis and cytokinesis of trypanosomes. EMBO J. 2003;22: 5336–5346. 10.1093/emboj/cdg518 14532107 PMC213772

[83] Zhou Q, Liu B, Sun Y, He CY. A coiled-coil- and C2-domain-containing protein is required for FAZ assembly and cell morphology in Trypanosoma brucei. J Cell Sci. 2011;124: 3848–3858. 10.1242/jcs.087676 22114307

[84] Sunter JD, Benz C, Andre J, Whipple S, McKean PG, Gull K, et al Flagellum attachment zone protein modulation and regulation of cell shape in Trypanosoma brucei life cycle transitions. J Cell Sci. 2015; jcs.171645. 10.1242/jcs.171645 PMC4541047 26148511

[85] Hayes P, Varga V, Olego-Fernandez S, Sunter J, Ginger ML, Gull K. Modulation of a cytoskeletal calpain-like protein induces major transitions in trypanosome morphology. J Cell Biol. 2014;206: 377–384. 10.1083/jcb.201312067 25092656 PMC4121973

[86] Hammarton TC, Monnerat S, Mottram JC. Cytokinesis in trypanosomatids. Curr Opin Microbiol. 2007;10: 520–527. 10.1016/j.mib.2007.10.005 18023244

[87] Ikeda KN, de Graffenried CL. Polo-like kinase is necessary for flagellum inheritance in Trypanosoma brucei. J Cell Sci. 2012;125: 3173–3184. 10.1242/jcs.101162 22427687

[88] Selvapandiyan A, Kumar P, Morris JC, Salisbury JL, Wang CC, Nakhasi HL. Centrin1 is required for organelle segregation and cytokinesis in Trypanosoma brucei. Mol Biol Cell. 2007;18: 3290–3301. 10.1091/mbc.E07-01-0022 17567955 PMC1951761

[89] LaCount DJ, Barrett B, Donelson JE. Trypanosoma brucei FLA1 is required for flagellum attachment and cytokinesis. J Biol Chem. 2002;277: 17580–17588. 10.1074/jbc.M200873200 11877446

[90] Sunter JD, Varga V, Dean S, Gull K. A dynamic coordination of flagellum and cytoplasmic cytoskeleton assembly specifies cell morphogenesis in trypanosomes. J Cell Sci. 2015;128: 1580–1594. 10.1242/jcs.166447 25736289 PMC4406125

[91] Absalon S, Kohl L, Branche C, Blisnick T, Toutirais G, Rusconi F, et al Basal body positioning is controlled by flagellum formation in Trypanosoma brucei. PloS One. 2007;2: e437 10.1371/journal.pone.0000437 17487282 PMC1857822

[92] Vaughan S, Kohl L, Ngai I, Wheeler RJ, Gull K. A repetitive protein essential for the flagellum attachment zone filament structure and function in Trypanosoma brucei. Protist. 2008;159: 127–136. 10.1016/j.protis.2007.08.005 17945531

[93] Davidge JA, Chambers E, Dickinson HA, Towers K, Ginger ML, McKean PG, et al Trypanosome IFT mutants provide insight into the motor location for mobility of the flagella connector and flagellar membrane formation. J Cell Sci. 2006;119: 3935–3943. 10.1242/jcs.03203 16954145

[94] Wheeler RJ, Scheumann N, Wickstead B, Gull K, Vaughan S. Cytokinesis in Trypanosoma brucei differs between bloodstream and tsetse trypomastigote forms: implications for microtubule-based morphogenesis and mutant analysis. Mol Microbiol. 2013;90: 1339–1355. 10.1111/mmi.12436 24164479 PMC4159584

[95] Viswanadha R, Hunter EL, Yamamoto R, Wirschell M, Alford LM, Dutcher SK, et al The ciliary inner dynein arm, I1 dynein, is assembled in the cytoplasm and transported by IFT before axonemal docking. Cytoskelet Hoboken NJ. 2014;71: 573–586. 10.1002/cm.21192 PMC4551456 25252184

[96] Alsford S, Turner DJ, Obado SO, Sanchez-Flores A, Glover L, Berriman M, et al High-throughput phenotyping using parallel sequencing of RNA interference targets in the African trypanosome. Genome Res. 2011;21: 915–924. 10.1101/gr.115089.110 21363968 PMC3106324

[97] Yamamoto R, Song K, Yanagisawa H-A, Fox L, Yagi T, Wirschell M, et al The MIA complex is a conserved and novel dynein regulator essential for normal ciliary motility. J Cell Biol. 2013;201: 263–278. 10.1083/jcb.201211048 23569216 PMC3628515

