# Supplementary figures and images for: Knockdown of Inner Arm Protein IC138 in Trypanosoma brucei Causes Defective Motility and Flagellar Detachment
Source: PLoS One. 2015 Nov 10;10(11):e0139579. doi: 10.1371/journal.pone.0139579 (PMC4640498; doi:10.1371/journal.pone.0139579)

S1 Figure

A

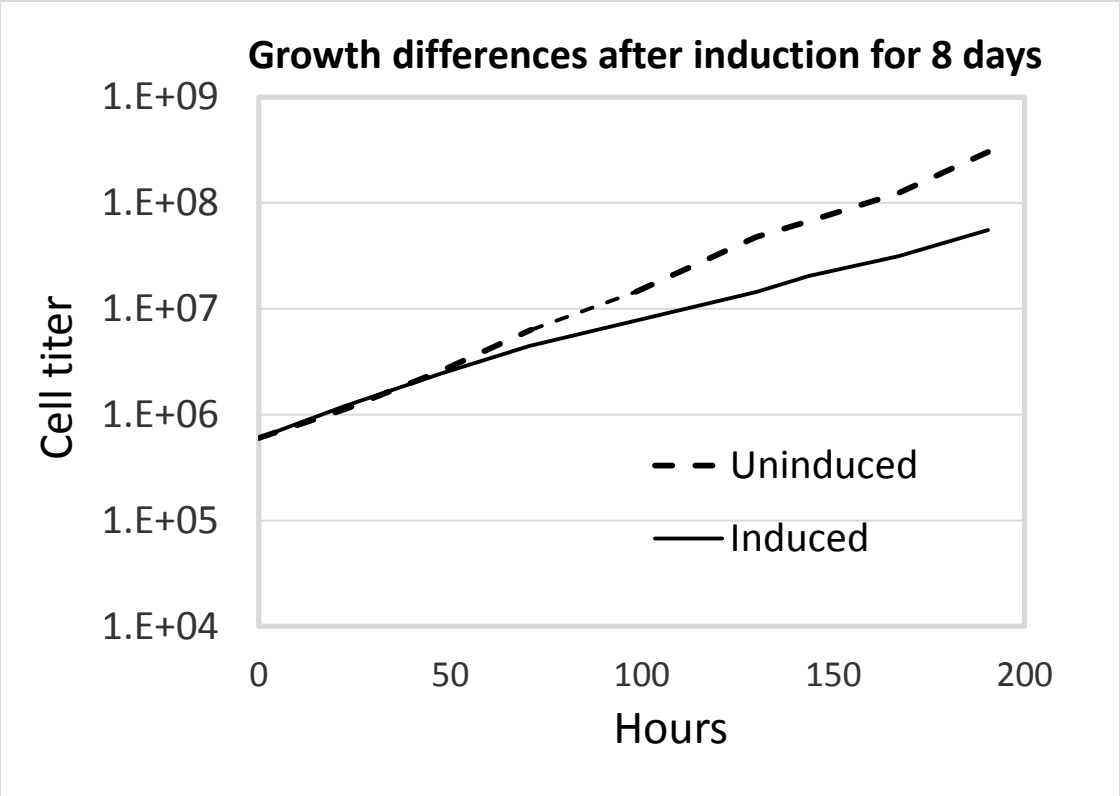

B

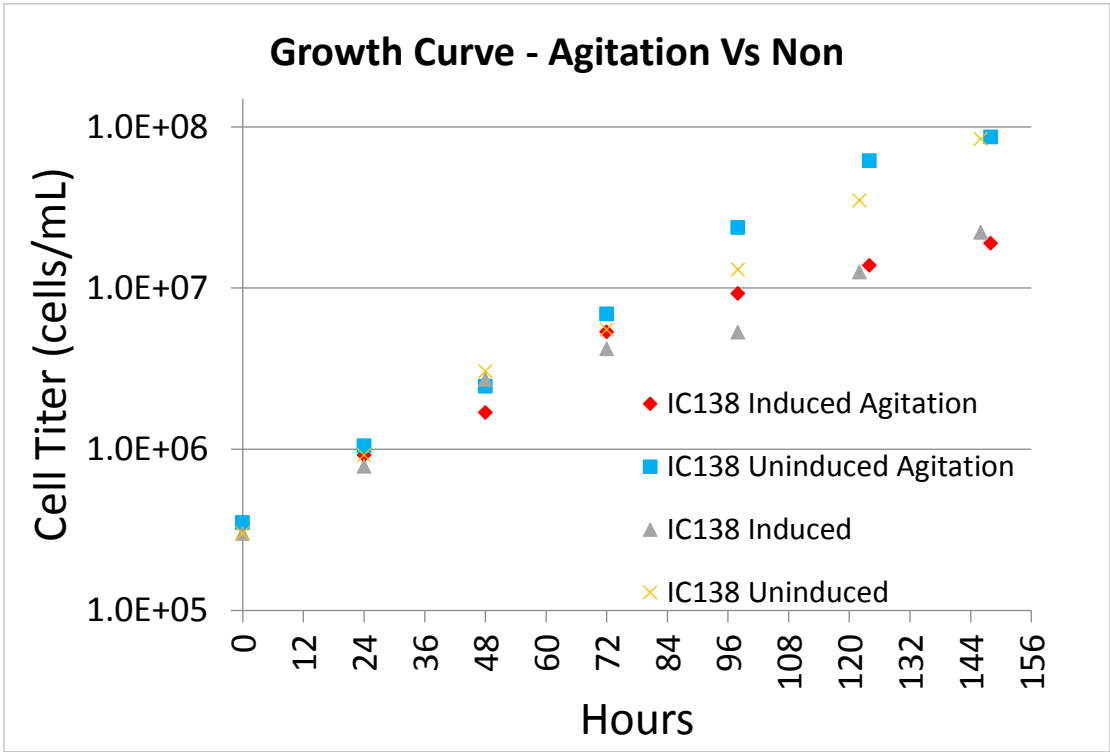

Supplement: S1 Fig — For agitation, flasks were incubated on an orbital shaker at 80 rpm for the duration of the induction. (PDF) [file pone.0139579.s001.pdf]

## S3 Figure

### A Cell motility of Uninduced cells

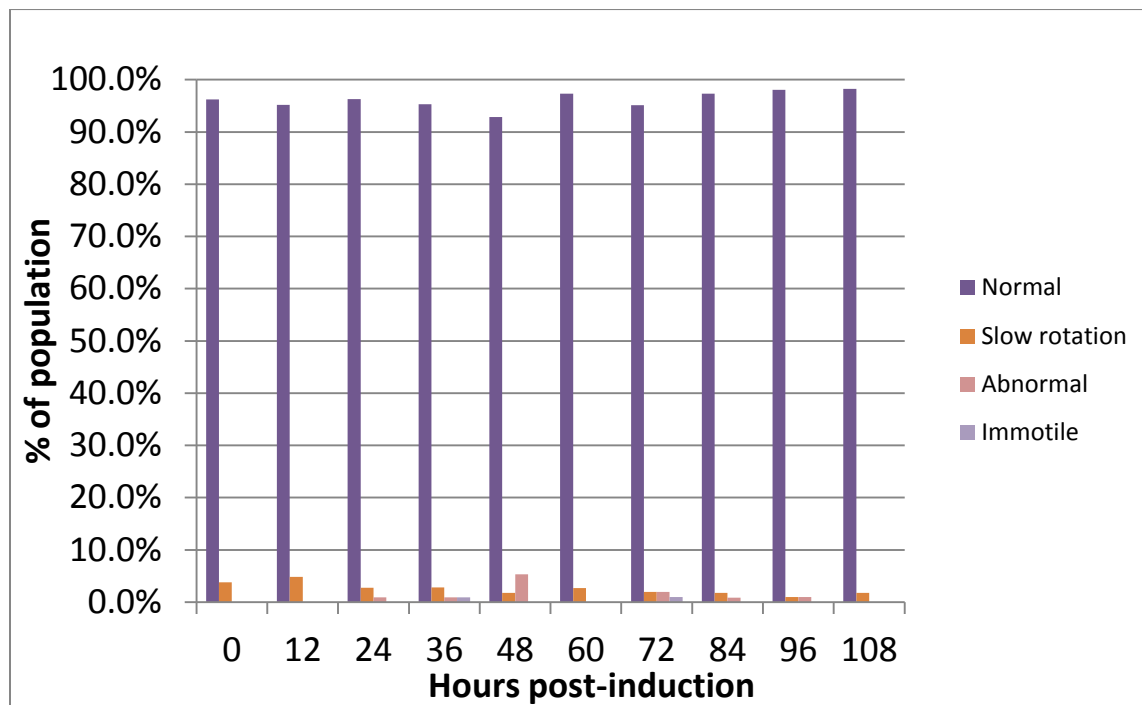

### B Flagellar Attachment of Uninduced Cells

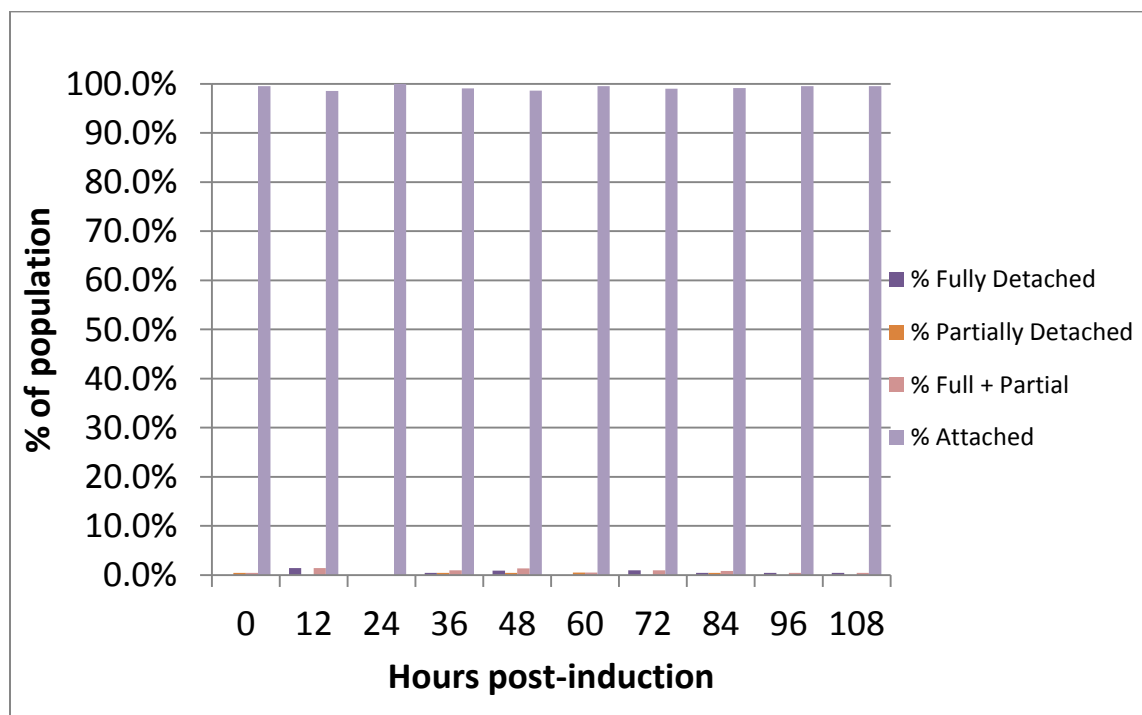

Supplement: S3 Fig — Scoring for cell motility phenotypes (A) and for flagellar detachment phenotypes (B). (PDF) [file pone.0139579.s003.pdf]

A

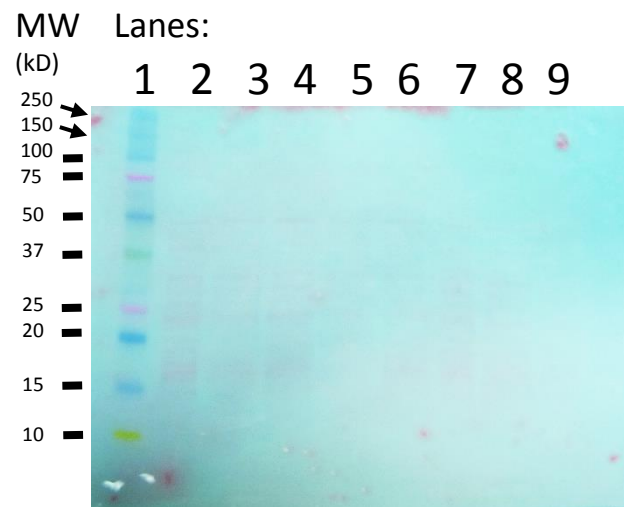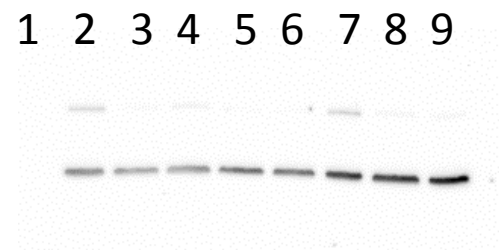

B

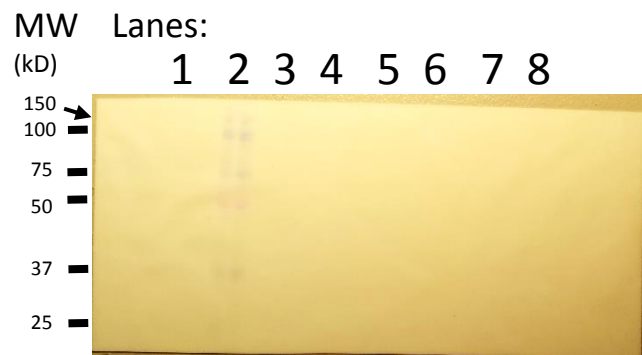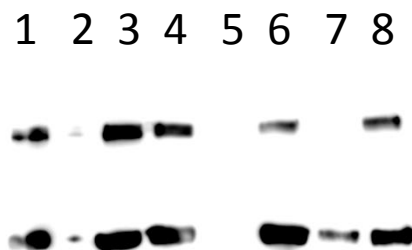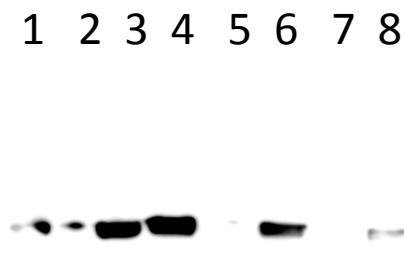

C

MW (kD) Lanes:

250  
150  
100  
75  
50  
37  
25

1 2 3 4 5 6 7 8 9 10

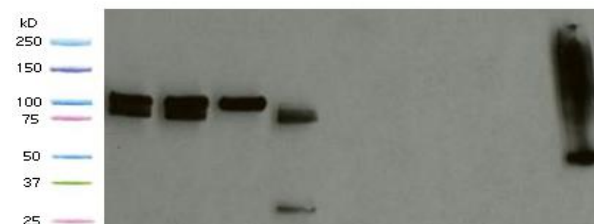

Supplement: S5 Fig — A. Paired images of blot used for knockdown quantification (Fig 1A). At left, a photograph of Ponceau-stained blot with pre-stained molecular weight markers indicated and at right, chemiluminescent signal from digital imager. Lanes are as follows: 1: Molecular weight marker, 2: Uninduced, 3: 48 H (replicate 1), 4: 48 H (repl. 2), 5: 72 H (repl. 1), 6: 72 H (repl. 2), 7: Uninduced, 8: 48 H (repl. 1), 9: 72 H (repl. 1). B. Matched images of blot used for detection of IC138::3xHA in fractions (Fig 1C), a photograph of Ponceau-stained blot with pre-stained molecular weight markers visible (left), chemiluminescent signal from immunoblot using anti-HA and anti-tubulin (center) and with anti-trypanin (right) from digital imager. Lanes are as follows: 1: Positive control for IC138::3xHA signal, 2: Molecular weight marker, 3: WC, 4: CY, 5: S1, 6: P1, 7: S2, 8: P2. C. Scanned image of film used for detection of IC138::3xHA in both IC138::3xHA strain and 29–13 negative control strain. Location of markers indicated after alignment of blot and film. Lanes are as follows: 1: Molecular weight marker, 2–5: IC138::3xHA in HS, LS, CY and WC fractions, respectively. 6–9: Wild type strain 29–13 in HS, LS, CY, and WC, respectively. 10: Positive control for HA epitope. (PDF) [file pone.0139579.s005.pdf]
