# Supplementary material for: Knockdown of Inner Arm Protein IC138 in Trypanosoma brucei Causes Defective Motility and Flagellar Detachment
Source: PLoS One. 2015 Nov 10;10(11):e0139579. doi: 10.1371/journal.pone.0139579 (PMC4640498; doi:10.1371/journal.pone.0139579)

S2 Figure

A. Nucleus: Kinetoplast ratios

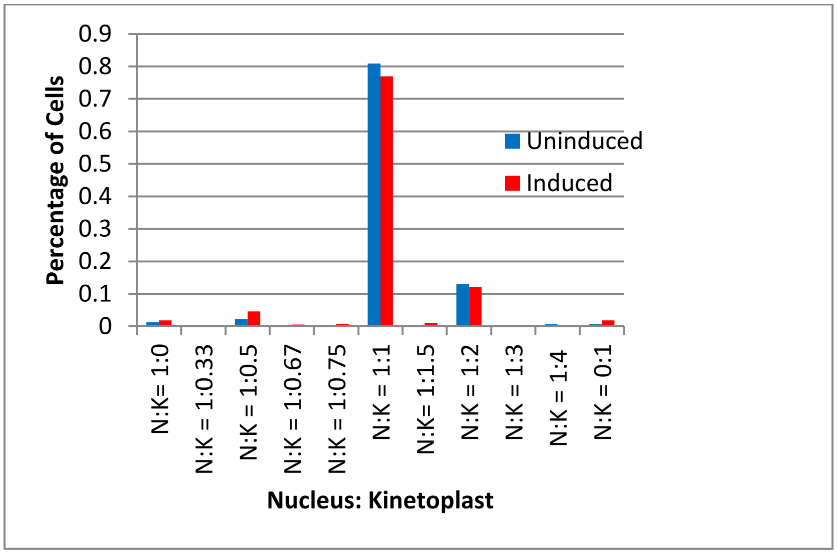

B. Relative Location of Kinetoplast, including frequency of anterior localization

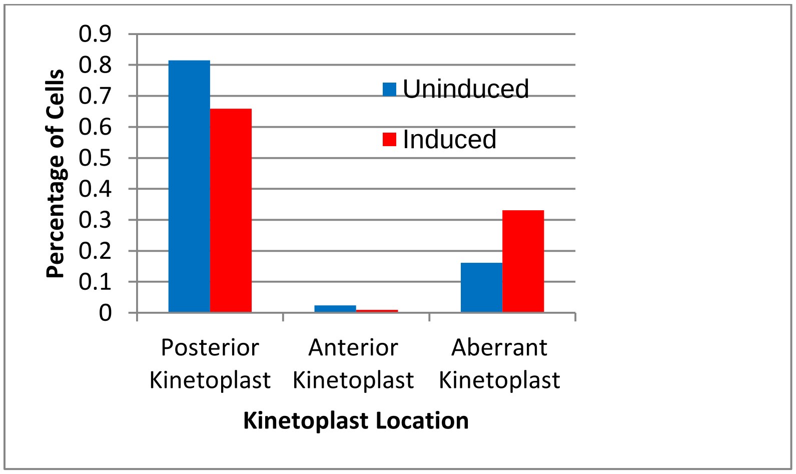

Supplement: S2 Fig — N:K classes (A) and location of kinetoplasts, including frequency of anterior localization (B). (PDF) [file pone.0139579.s002.pdf]
