# Supplementary material for: Knockdown of Inner Arm Protein IC138 in Trypanosoma brucei Causes Defective Motility and Flagellar Detachment
Source: PLoS One. 2015 Nov 10;10(11):e0139579. doi: 10.1371/journal.pone.0139579 (PMC4640498; doi:10.1371/journal.pone.0139579)

S4 Figure

A. Motility of RNAi-induced cells with partially or fully detached flagella

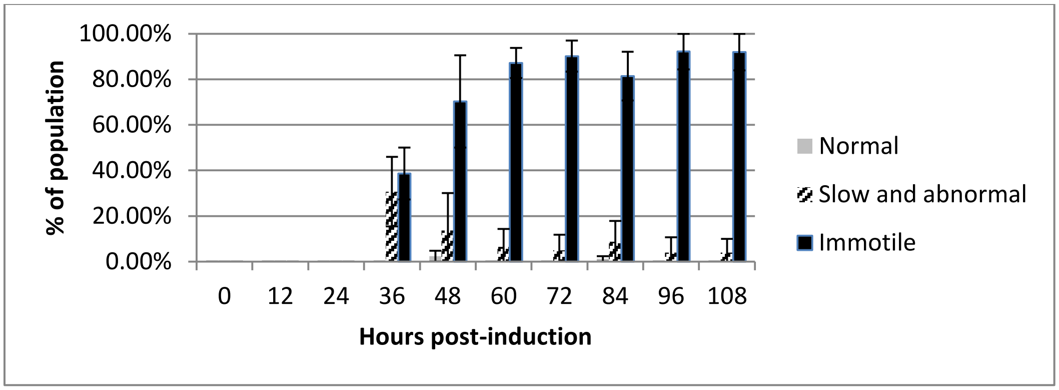

B. Motility: cells with attached flagella only

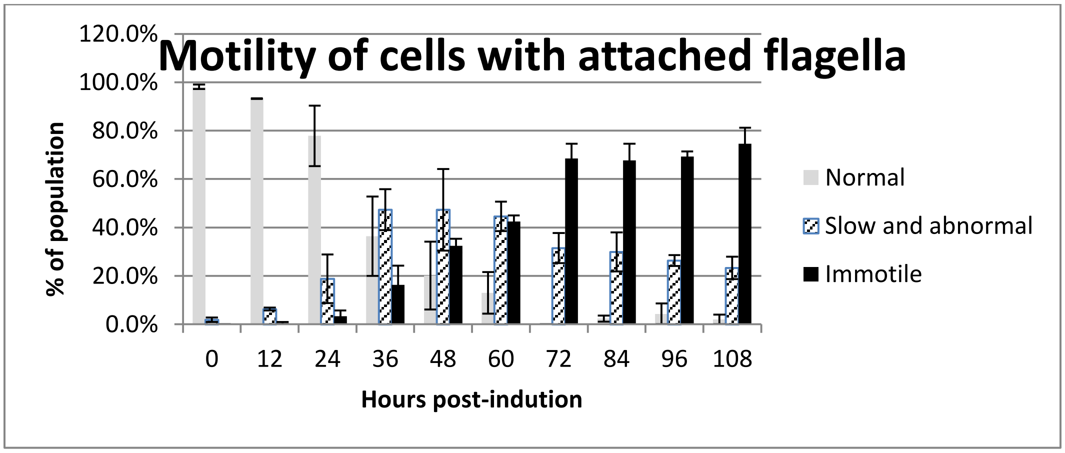

Supplement: S4 Fig — Motility in IC138RNAi induced cells that have partially or fully detached flagella (A) and cell body movement in IC138RNAi induced cells, only those with completely attached flagella (B). (PDF) [file pone.0139579.s004.pdf]
