# Supplementary material for: Knockdown of Inner Arm Protein IC138 in Trypanosoma brucei Causes Defective Motility and Flagellar Detachment
Source: PLoS One. 2015 Nov 10;10(11):e0139579. doi: 10.1371/journal.pone.0139579 (PMC4640498; doi:10.1371/journal.pone.0139579)
Supplement: S1 Table — (PDF) [file pone.0139579.s006.pdf]

Date:	Time:	Cells:	Hours Induced	
	**Fully Attached Flagella**	**Partially Detached Flagella (Loop)**	**Partially Detached Flagella (Loop and tail)**	**Fully Detached Flagella**
**Normal Body Movement**	Normal	Normal	Normal	Normal
	Abnormal	Abnormal	Abnormal	Abnormal
	Paralyzed	Partially Paralyzed	Partially Paralyzed	Paralyzed
		Paralyzed	Paralyzed	
**Slow Helical Body Movement**	Normal	Normal	Normal	Normal
	Abnormal	Abnormal	Abnormal	Abnormal
	Paralyzed	Partially Paralyzed	Partially Paralyzed	Paralyzed
		Paralyzed	Paralyzed	
**Abnormal Body Movement "Twitchy"**	Normal	Normal	Normal	Normal
	Abnormal	Abnormal	Abnormal	Abnormal
	Paralyzed	Partially Paralyzed	Partially Paralyzed	Paralyzed
		Paralyzed	Paralyzed	
**Immotile Body (Tail Twitch)**	Normal	Normal	Normal	Normal
	Abnormal	Abnormal	Abnormal	Abnormal
	Paralyzed	Partially Paralyzed	Partially Paralyzed	Paralyzed
		Paralyzed	Paralyzed	

Cannot score:

Clumps:
