# Supplementary material for: Knockdown of Inner Arm Protein IC138 in Trypanosoma brucei Causes Defective Motility and Flagellar Detachment
Source: PLoS One. 2015 Nov 10;10(11):e0139579. doi: 10.1371/journal.pone.0139579 (PMC4640498; doi:10.1371/journal.pone.0139579)
Supplement: S2 Table — (DOCX) [file pone.0139579.s007.docx]

S2 Table. Description of phenotypic characteristics of IC138 RNAi knockdown cultures

| **Detachment Phenotypes** | | **Description** |
| --- | --- | --- |
| Fully Attached Flagella | | Attached all along the cell body |
| Partially Detached Flagella^a^ | | One or more fragments disconnected along cell body |
| Fully Detached Flagella | | Detached along cell body except at flagellar pocket |
| **Motility Phenotypes^a^** | | **Description** |
| Body | Normal | Normal movement compared to uninduced sample |
|  | Slow Rotation | Slower than uninduced sample but retain rotational motion |
|  | Abnormal | Twitchy, jerking or pulsating movement of the cell body |
|  | Immotile | No movement of the cell body |
| Flagellum | Normal | Normal movement compared to uninduced sample |
|  | Abnormal | Unnatural movement of flagellum compared to uninduced sample; twitchy or slow |
|  | Partially Paralyzed | Detached portion of flagellum paralyzed but attached portion continues to move (only in partially detached flagella) |
|  | Paralyzed | No movement of the flagellum |

^a^ For partially detached flagella, a further distinction was made between flagella which were detached only along a central portion, and so appeared as a “loop” off of the cell body and those that appeared to have both a loop and a detached tip (called ”loop and tail”). However these two categories were combined for analysis purposes.

^b^ For motility, cells were scored based on their body movement (starting at the wide base of the cell and extending about 3/4 of the way to the tip) and their flagellar movement (the anterior 1/4 of the cell with an attached flagellum or the movement of any detached portions).
