# Supplementary material for: Knockdown of Inner Arm Protein IC138 in Trypanosoma brucei Causes Defective Motility and Flagellar Detachment
Source: PLoS One. 2015 Nov 10;10(11):e0139579. doi: 10.1371/journal.pone.0139579 (PMC4640498; doi:10.1371/journal.pone.0139579)
Supplement: S4 Table — (PDF) [file pone.0139579.s009.pdf]

S4 Table: Nucleus: Cell ratios

	Uninduced			induced		
	Cells	Nuclei	ratio	Cells	Nuclei	
	25	27	1.08	55	70	1.27
	34	38	1.12	61	70	1.15
	43	48	1.12	55	64	1.16
	30	33	1.10	72	88	1.22
	39	44	1.13	53	60	1.13
	55	66	1.20	24	32	1.33
	43	45	1.05	36	47	1.31
	55	69	1.25	22	23	1.05
				17	20	1.18
Total	324	370		395	474	
Mean			1.13			1.20
St. Dev.			**0.06**			**0.09**
